# Simultaneous Dual Selective Targeted Delivery of Two Covalent Gemcitabine Immunochemotherapeutics and Complementary Anti-Neoplastic Potency of [Se]-Methylselenocysteine

**DOI:** 10.4236/jct.2015.61009

**Published:** 2015-01-19

**Authors:** C. P. Coyne, Toni Jones, Ryan Bear

**Affiliations:** 1 Department of Basic Sciences, College of Veterinary Medicine, Mississippi State University, Mississippi State, USA; 2 Wise Center, Mississippi State University, Mississippi State, USA

**Keywords:** Gemcitabine, Anti-EGFR, Anti-HER2/*neu*, Covalent Immunochemotherapeutic, Gemcitabine-(C_4_-*amide*)-[Anti-EGFR], Gemcitabine-(C_4_-*amide*)-[Anti-HER2/*neu*], Mammary Adenocarcinoma (SKBr-3), [Se]-Methylselenocysteine

## Abstract

The anti-metabolite chemotherapeutic, gemcitabine is relatively effective for a spectrum of neoplastic conditions that include various forms of leukemia and adenocarcinoma/carcinoma. Rapid systemic deamination of gemcitabine accounts for a brief plasma half-life but its sustained administration is often curtailed by sequelae and chemotherapeutic-resistance. A molecular strategy that diminishes these limitations is the molecular design and synthetic production of covalent gemcitabine immunochemotherapeutics that possess properties of selective “targeted” delivery. The simultaneous dual selective “targeted” delivery of gemcitabine at two separate sites on the external surface membrane of a single cancer cell types represents a therapeutic approach that can increase cytosol chemotherapeutic deposition; prolong chemotherapeutic plasma half-life (reduces administration frequency); minimize innocent exposure of normal tissues and healthy organ systems; and ultimately enhance more rapid and thorough resolution of neoplastic cell populations. Materials and Methods: A light-reactive gemcitabine intermediate synthesized utilizing succinimidyl 4,4-azipentanoate was covalently bound to anti-EGFR or anti-HER2/*neu* IgG by exposure to UV light (354-nm) resulting in the synthesis of covalent immunochemotherapeutics, gemcitabine-(C_4_-*amide*)-[anti-EGFR] and gemcitabine-(C_4_-*amide*)-[anti-HER2/*neu*]. Cytotoxic anti-neoplastic potency of gemcitabine-(C_4_-*amide*)-[anti-EGFR] and gemcitabine-(C_4_-*amide*)-[anti-HER2/*neu*] between gemcitabine-equivalent concentrations of 10^−12^ M and 10^−6^ M was determined utilizing chemotherapeutic-resistant mammary adenocarcinoma (SKRr-3). The organoselenium compound, [Se]-methylselenocysteine was evaluated to determine if it complemented the anti-neoplastic potency of the covalent gemcitabine immunochemotherapeutics. Results: Gemcitabine-(C_4_-*amide*)-[anti-EGFR], gemcitabine-(C_4_-*amide*)-[anti-HER2/*neu*] and the dual simultaneous combination of gemcitabine-(C_4_-*amide*)-[anti-EGFR] with gemcitabine-(C_4_-*amide*)-[anti-HER2/*neu*] all had anti-neoplastic cytotoxic potency against mammary adenocarcinoma. Gemcitabine-(C_4_-*amide*)-[anti-EGFR] and gemcitabine-(C_4_-*amide*)-[anti-HER2/*neu*] produced progressive increases in anti-neoplastic cytotoxicity that were greatest between gemcitabine-equivalent concentrations of 10^−9^ M and 10^−6^ M. Dual simultaneous combinations of gemcitabine-(C_4_-*amide*)-[anti-EGFR] with gemcitabine-(C_4_-*amide*)-[anti-HER2/*neu*] produced levels of anti-neoplastic cytotoxicity intermediate between each of the individual covalent gemcitabine immunochemotherapeutics. Total anti-neoplastic cytotoxicity of the dual simultaneous combination of gemcitabine-(C_4_-*amide*)-[anti-EGFR] and gemcitabine-(C_4_-*amide*)-[anti-HER2/*neu*] against chemotherapeutic-resistant mammary adenocarcinoma (SKBr-3) was substantially higher when formulated with [Se]-methylsele-nocysteine.

## 1. Introduction

Monoclonal immunoglobulin preparations or pharmaceuticals with binding-avidity for HER2/*neu* (e.g. anti-HER2/*neu*: trastuzumab, pertuzumab), EGFR (e.g. anti-EGFR: cetuximab, gefitinib) [1]–[4], HER2/*neu* and EGFR (e.g. anti-HER2/*neu* and anti-EGFR: panitumumab) [3]–[6] IGF-1R, VEGFR and inhibitors of trophic membrane receptors can all potentially be effective treatment options for certain neoplastic conditions including cancer affecting the breast, intestinal tract, lung or prostate. The significant advantage of these preparations is their ability to function as a selective anti-cancer treatment modality that also avoids many of the sequelae associated with conventional chemotherapy. Unfortunately, most monoclonal immunoglobulin-based therapies that inhibit the function of trophic membrane receptors are usually only capable of exerting cytostatic properties and as a monotherapy are almost invariably plagued by an inability to evoke cytotoxic activity that is potent enough to effectively resolve most aggressive and advanced forms of neoplastic disease [7]–[12]. Alternatively, enhanced levels of anti-neoplastic cytotoxicity can be attained when monoclonal immunoglobulin-based biotherapies are applied in concert with conventional chemotherapeutics or other anti-cancer treatment modalities [13]–[15].

The potential for selective and simultaneous “targeted” delivery of a single or multiple chemotherapeutic agents or pharmaceuticals at two or more uniquely or over-expressed trophic receptor complexes for the purpose of evoking an enhanced level of anti-neoplastic cytotoxicity or other types of a biological effect against specific cancer cell types remains a facet of oncology and pharmacology that has not been extensively delineated. Based on the increased level of anti-neoplastic cytotoxicity that can potentially be gained through dual simultaneous selectively targeted” epirubicin delivery at trophic receptors over-expressed (EGFR) and highly over-expressed (HER2/*neu*) by chemotherapeutic resistant mammary adenocarcinoma (SKBr-3) [16] the concept of this molecular strategy does have therapeutic merit. Reported in this research investigation is the anti-neoplastic cytotoxicity of gemcitabine-(C_4_-*amide*)-[anti-EGFR] and gemcitabine-(C_4_-*amide*)-[anti-HER2/*neu*] against chemotherapeutic-resistant mammary adenocarcinoma (SKBr-3) applied simultaneously as a dual selectively “targeted” chemotherapeutic regimen. The strategy has clinical relevance in part due to the effectiveness of gemcitabine, especially in combination with paclitaxel, carboplatin and cisplatin following anthracycline failure in the treatment of metastatic breast cancer [17]. The objective of the research investigations was to determine if simultaneous selective “targeted” delivery of two covalent gemcitabine immunochemotherapeutics is possible at two different endogenous trophic receptor sites over-expressed on the surface membrane of a neoplastic cell type and establish the potential for [Se]-methylselenocysteine to complement the anti-cancer cytotoxic potency attained with this molecular strategy.

## 2. Materials and Methods

### 2.1. Covalent Gemcitabine Immunochemotherapeutic Synthesis

#### Phase-I Synthesis Scheme for UV-Photoactivated Chemotherapeutic Intermediates

**Figure 1:**
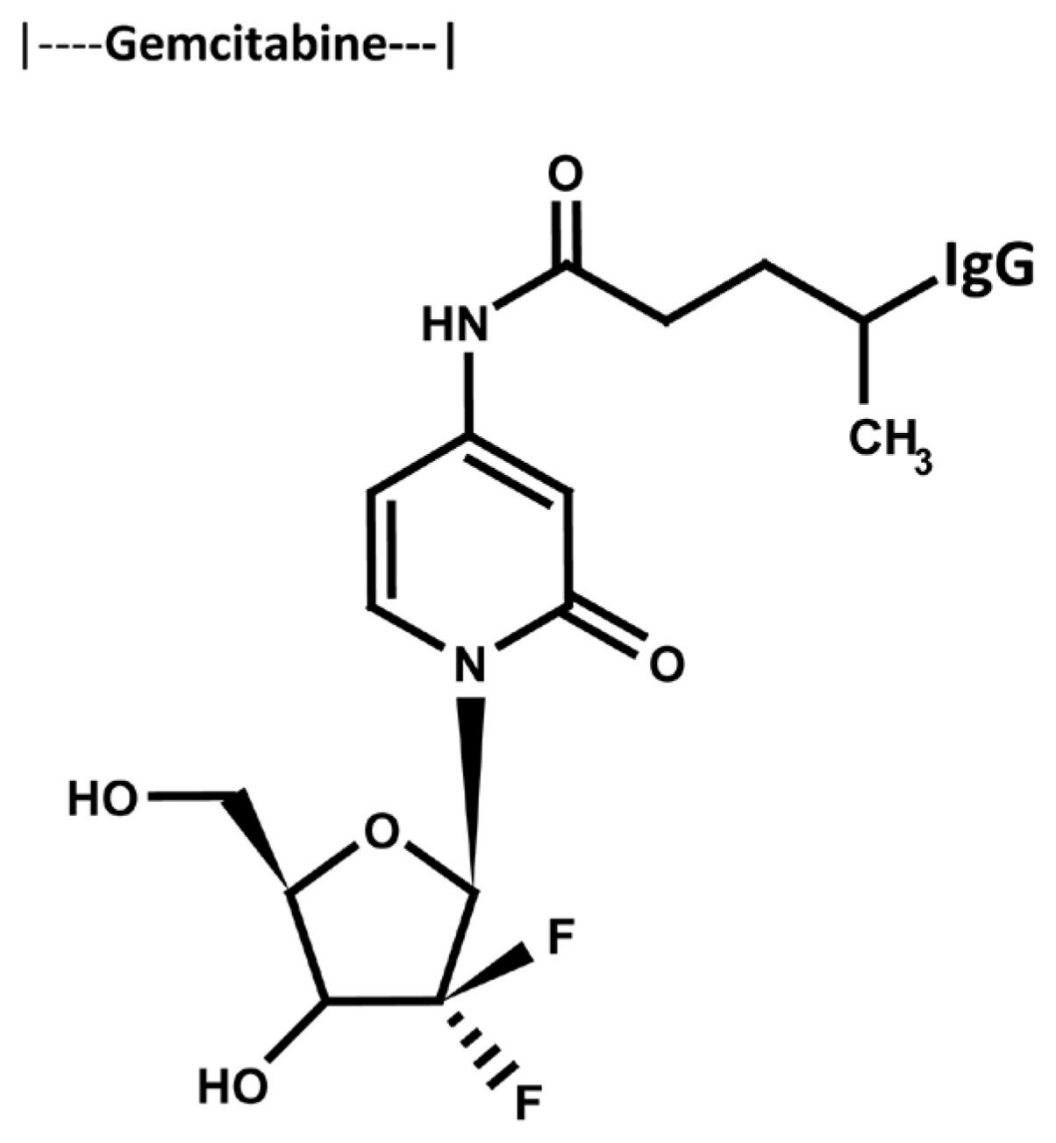
Molecular design and chemical structure of the covalent immunochemotherapeutics, gemcitabine-(C_4_-*amide*)-[anti-HER2/*neu*] and gemcitabine-(C_4_-*amide*)-[anti-EGFR] synthesized utilizing a 2-stage organic chemistry reaction scheme that initially generates a gemcitabine UV-photoactivated intermediate. A synthetic covalent bond formed at the C_4_ cytosine-like mononamine group of gemcitabine chemotherapeutic and the side chains of amino acid residues within the sequence of immunoglobulin fractions.

The cytosine-like C_4_-monoamine of gemcitabine (0.738 mg, 2.80 × 10^−3^ mmoles) was reacted at a 2.5:1 molar-ratio with the amine-reactive *N-*hydroxysuccinimide ester “leaving” complex of succinimidyl 4,4-azipentanoate (0.252 mg, 1.12 × 10^−3^ mmoles) in the presence of triethylamine (TEA: 50 mM final concentration) utilizing dimethylsulfoxide as an anhydrous organic solvent system (Figure 1) [18] [19]. The reaction mixture formulated from stock solutions of gemcitabine and succinimidyl 4,4-azipentanoate was continually stirred gently at 25°C over a 4-hour incubation period in the dark and protected from exposure to light. The relatively long incubation period of 4 hours was utilized to maximize degradation of the ester group of any residual succinimidyl 4,4-azipentanoate that may not of reacted during the first 30 to 60 minutes with the C_4_ cytadine-like monoamine group of gemcitabine.

#### Phase-II Synthesis Scheme for Covalent Gemcitabine Immunochemotherapeutics Utilizing a UV-Photoactivated Chemotherapeutic Intermediate

Immunoglobulin fractions of anti-HER2/*neu* or anti-EGFR (1.5 mg, 1.0 × 10^−5^ mmoles) in buffer (PBS: phosphate 0.1, NaCl 0.15 M, EDTA 10 mM, pH 7.3) were combined at a 1:10 molar-ratio with the UV-photoactivated gemcitabine-(C_4_-*amide*) intermediate (*Phase-*1 *end product*) and allowed to gently mix by constant stirring for 5 minutes at 25°C in the dark [19]. The photoactivated group of the gemcitabine-(C_4_-*amide*) intermediate was reacted with groups associated with the side chains of amino acid residues within the sequence of anti-EGFR or anti-HER2/*neu* monoclonal immunoglobulins during a 15 minute exposure to UV light at 354-nm (reagent activation range 320 – 370 nm) in combination with constant gentle stirring (Figure 1). Residual gemcitabine was removed from the covalent gemcitabine immunochemotherapeutics by microscale column chromatography following PBS pre-equilibration of media (phosphate 0.1 M, NaCl 0.15 M, pH 7.3).

### 2.2. Molecular Analysis and Characterization of Properties

#### General Analysis

Quantitation of the amount of non-covalently bound gemcitabine contained within covalent gemcitabine-(C_4_-*amide*)-[anti-HER2/*neu*] and gemcitabine-(C_4_-*amide*)-[anti-EGFR] immunochemotherapeutics following separation by column chromatography was determined by measuring absorbance at 265 – 268 nm [20] [21] for the resulting supernatant following precipitation of covalent gemcitabine-immunochemotherapeutics with methanol:acetonitrile (1:9 v/v). It is also possible to calculate the amount of gemcitabine covalent incorporated into the immunochemotherapeutics by measuring residual unbound gemcitabine before and after the Phase II reaction [22]–[24].

Determination of the immunoglobulin concentration for covalent gemcitabine-(C_4_-*amide*)-[anti-HER2/*neu*] and gemcitabine-(C_4_-*amide*)-[anti-EGFR] immunochemotherapeutics was determined by measuring absorbance at 280 nm in combinations with utilizing a 235 nm-vs-280 nm standardized reference curve in order to accommodate for any potential absorption profile over-lap at 280 nm between immunoglobulin and the gemcitabine moiety.

#### Mass-Separation Analysis for Detection of Polymerization and Fragmentation

Covalent gemcitabine-(C_4_-*amide*)-[anti-EGFR] and gemcitabine-(C_4_-*amide*)-[anti-HER2/*neu*] immunochemotherapeutics in addition to reference control anti-EGFR and anti-HER2/*neu* immunoglobulin fractions were adjusted to a standardized protein concentration of 60 μg/ml and then combined 50/50 v/v with conventional SDS-PAGE sample preparation buffer (Tris/glycerol/bromphenyl blue/SDS) formulated without 2-mercaptoethanol or boiling. Each covalent immunochemotherapeutic, the reference control immunoglobulin fraction (0.9 μg/well) and a mixture of pre-stained reference control molecular weight markers were then developed by non-reducing SDS-PAGE (11% acrylamide) performed at 100 V constant voltage at 3°C for 2.5 hours.

#### Immunodetection Analyses for Polymerization and Fragmentation Detection

Covalent gemcitabine-(C_4_-*amide*)-[anti-HER2/*neu*] and gemcitabine-(C_4_-*amide*)-[anti-EGFR] immunochemotherapeutics following mass/size-dependent separation by non-reducing SDS-PAGE were equilibrated in tank buffer devoid of methanol. Mass/size-separated gemcitabine-[anti-HER2/*neu*] immunochemotherapeutics contained within acrylamide SDS-PAGE gels were then transferred laterally onto sheets of nitrocellulose membrane at 20 volts (constant voltage) for 16 hours at 2°C to 3°C (Note: n = 2 locations) with the transfer manifold packed in crushed ice.

Nitrocellulose membranes with laterally-transferred covalent gemcitabine immunochemotherapeutics were then equilibrated in Tris buffered saline (TBS: Tris HCl 0.1 M, NaCl 150 mM, pH 7.5, 40 ml) at 4°C for 15 minutes followed by incubation in TBS blocking buffer solution (Tris 0.1 M, pH 7.4, 40 ml) containing bovine serum albumin (5%) for 16 hours at 2°C to 3°C applied in combination with gentle horizontal agitation. Prior to further processing, nitrocellulose membranes were vigorously rinsed in Tris buffered saline (Tris 0.1 M, pH 7.4, 40 ml, n = 3x).

Rinsed BSA-blocked nitrocellulose membranes developed for Western-blot (immunodetection) analyses were incubated with biotinylated goat anti-murine IgG (1:10,000 dilution) at 4°C for 18 hours applied in combination with gentle horizontal agitation. Nitrocellulose membranes were then vigorously rinsed in TBS (pH 7.4, 4°C, 50 ml, n = 3) followed by incubation in blocking buffer (Tris 0.1 M, pH 7.4, with BSA 5%, 40 ml). Blocking buffer was decanted from nitrocellulose membrane blots which were then rinsed in TBS (pH 7.4, 4°C, 50 ml, n = 3) before incubation with HRPO-strepavidin (1:100,000 dilution) at 4°C for 2 hours applied in combination with gentle horizontal agitation. Prior to chemiluminescent development nitrocellulose membranes were vigorously rinsed in Tris buffered saline (Tris 0.1 M, pH 7.4, 40 ml, n = 3). Following development with conjugated HRPO-strepavidin the nitrocellulose membranes were then incubated with HRPO chemiluminescent substrate (25°C; 5-to-10 minutes). Chemiluminescent autoradiography images were acquired by exposing radiographic film (Kodak BioMax XAR) to nitrocellulose membranes sealed within transparent ultra-clear re-sealable plastic bags.

### 2.3. Mammary Adenocarcinoma: Neoplastic Disease *ex-Vivo* Model

#### Mammary Adenocarcinoma Tissue Culture Cell Culture

The human mammary adenocarcinoma (SKBr-3) was utilized as an *ex-vivo* model for neoplastic disease. Populations of the mammary adenocarcinoma (SKBr-3) were propagated at ≥85% level of confluency in 150-cc^2^ tissue culture flasks containing McCoy’s 5a Modified Medium supplemented with fetal bovine serum (10% v/v) and penicillin-streptomycin at a temperature of 37°C under a gas atmosphere of air (95%) and carbon dioxide (5% CO_2_). Trypsin or any other biochemically active enzyme fraction were not used to facilitate harvest of mammary adenocarcinoma SKBr-3 cell suspensions for seeding of tissue culture flasks or multi-well tissue culture plates. Growth media was not supplemented with growth factors, growth hormones or any other type of growth stimulant.

Characteristic features and biological properties of the mammary adenocarcinoma (SKBr-3) cell line includes chemotherapeutic-resistance, over-expression of epidermal growth factor receptor 1 (EGFR, ErbB-1, HER1: at 2.2 × 10^5^/cell), and high over-expression of epidermal growth factor receptor 2 (EGFR2, HER2/*neu*, ErbB-2, CD340, p185: at 1 × 10^6^/cell).

#### Cell-ELISA Total Membrane-Bound Immunoglobulin Assay

Cell suspensions of mammary adenocarcinoma (SKBr-3) were seeded into 96-well microtiter plates in aliquots of 2 × 10^5^ cells/well and allowed to form a confluent adherent monolayer over a period of 48 hours. The growth media content in each individual well was removed manually by pipette and the cellular monolayers were then serially rinsed (n = 3) with PBS followed by their stabilization onto the plastic surface of 96-well plates with paraformaldehyde (4% in PBS, 15 minutes). Stabilized cellular monolayers were then incubated with covalent gemcitabine-(C_4_-*amide*)-[anti-HER2/*neu*] and gemcitabine-(C_4_-*amide*)-[anti-EGFR] immunochemotherapeutics formulated at gradient concentrations of 0.1, 0.25, 0.5, 1.0, 5.0 and 10 μg/ml in tissue culture growth media (200 μl/well). Direct contact incubation between (SKBr-3) cellular monolayers and gemcitabine-(C_4_-*amide*)-[anti-HER2/*neu*] and gemcitabine-(C_4_-*amide*)-[anti-EGFR] was performed at 37°C during an incubation period of 3-hours under a gas atmosphere of air (95%) and carbon dioxide (5% CO_2_). Following serial rinsing with PBS (n = 3), development of stabilized mammary adenocarcinoma (SKBr-3) monolayers entailed incubation with *β*-galactosidase conjugated goat anti-mouse IgG (1:500 dilution) for 2 hours at 25°C with residual unbound immunoglobulin removed by serial rinsing with PBS (n = 3). Final cell ELISA development required serial rinsing (n = 3) of stabilized (SKBr-3) monolayers with PBS followed by incubation with nitrophenyl-*β*-D-galactopyranoside substrate (100 μl/well of ONPG formulated fresh at 0.9 mg/ml in PBS pH 7.2 containing MgCl_2_ 10 mM, and 2-mercaptoethanol 0.1 M). Absorbance within each individual well was measured at 410 nm (630 nm reference wavelength) after incubation at 37°C for a period of 15 minutes.

#### Cell Vitality Stain-Based Assay for Measuring Anti-neoplastic cytotoxicity

Individual preparations of gemcitabine-(C_4_-*amide*)-[anti-HER2/*neu*] and gemcitabine-(C_4_-*amide*)-[anti-EGFR] were formulated in growth media at standardized gemcitabine-equivalent concentrations of 10^−10^, 10^−9^, 10^−8^, 10^−7^, and 10^−6^ M (final concentration). Each gemcitabine-equivalent concentration of the covalent immunochemotherapeutics were then transferred in triplicate into 96-well microtiter plates containing mammary adenocarcinoma (SKBr-3) monolayers and growth media (200 μl/well). Covalent immunochemotherapeutics where then incubated in direct contact with monolayer mammary adenocarcinoma (SKBr-3) populations for a period of 182-hours (37°C under a gas atmosphere of air (95%) and carbon dioxide/CO_2_ (5%). Following the initial 96-hour incubation period, mammary adenocarcinoma (SKBr-3) populations were replenished with fresh tissue culture media with or without covalent gemcitabine-immunochemotherapeutics or benzimidazole tubulin/microtubule inhibitors.

Anti-neoplastic cytotoxicity for gemcitabine-(C_4_-*amide*)-[anti-HER2/*neu*] and gemcitabine-(C_4_-*amide*)-[anti-EGFR] were measured by removing all contents within the 96-well microtiter plates manually by pipette followed by serial rinsing of monolayers (n = 3) with PBS followed by incubation with 3-[4,5-dimethylthiazol-2-yl]-2,5-diphenyl tetrazolium bromide vitality stain reagent formulated in RPMI-1640 growth media devoid of pH indicator or bovine fetal calf serum (MTT: 5 mg/ml). During an incubation period of 3 – 4 hours at 37°C under a gas atmosphere of air (95%) and carbon dioxide (5% CO_2_) the enzyme mitochondrial succinate dehydrogenase was allowed to convert the MTT vitality stain reagent to navy-blue formazone crystals within the cytosol of mammary adenocarcinoma (SKBr-3) populations (some reports suggest that NADH/NADPH dependent cellular oxidoreductase enzymes may also be involved in the biochemical conversion process). Contents were then removed from each of the 96-wells in the microtiter plate, followed by serial rinsing with PBS (n = 3). The resulting blue intracellular formazone crystals were dissolved with DMSO (300 μl/well) and then spectrophotometric absorbance of the resulting blue-colored supernatant measured at 570 nm using a computer-integrated microtiter plate reader.

## 3. Results

### 

#### Molar-Incorporation Index

Size-separation of gemcitabine-(C_4_-*amide*)-[anti-HER2/*neu*] and gemcitabine-(C_4_-*amide*)-[anti-EGFR] by microscale desalting/buffer exchange column chromatography consistently yields covalent immunochemotherapeutic preparations that contained <4.0% of residual chemotherapeutic that was not covalently bound to immunoglobulin [16] [18] [19] [24] [25]. Small residual amounts of non-covalently bound chemotherapeutic remaining within covalent immunochemotherapeutic preparations is generally accepted to not be available for further removal through any additional sequential column chromatography separations [26]. The calculated estimate of the molar-incorporation-index for the covalent gemcitabine-(C_4_-*amide*)-[IgG] immunochemotherapeutics was 2.78 utilizing the organic chemistry reaction scheme to form an amide bond at the C_4_ cytosine-like monoamine of gemcitabine and synthesis of the UV-photoactivated gemcitabine-(C_4_-*amide*) intermediate (Figure 1). The molar-incorporation-ration of 2.78-to-1 for gemcitabine-(C_4_-*amide*)-[anti-HER2/*neu*] and gemcitabine-(C_4_-*amide*)-[anti-EGFR] was relatively larger than the 1.1-to-1 gemcitabine molar-incorporation-index attained during the synthesis of gemcitabine-(C_5_-*methylcarbamate*)-[anti-HER2/*neu*] [24].

#### Molecular Weight Profile Analysis

**Figure 2:**
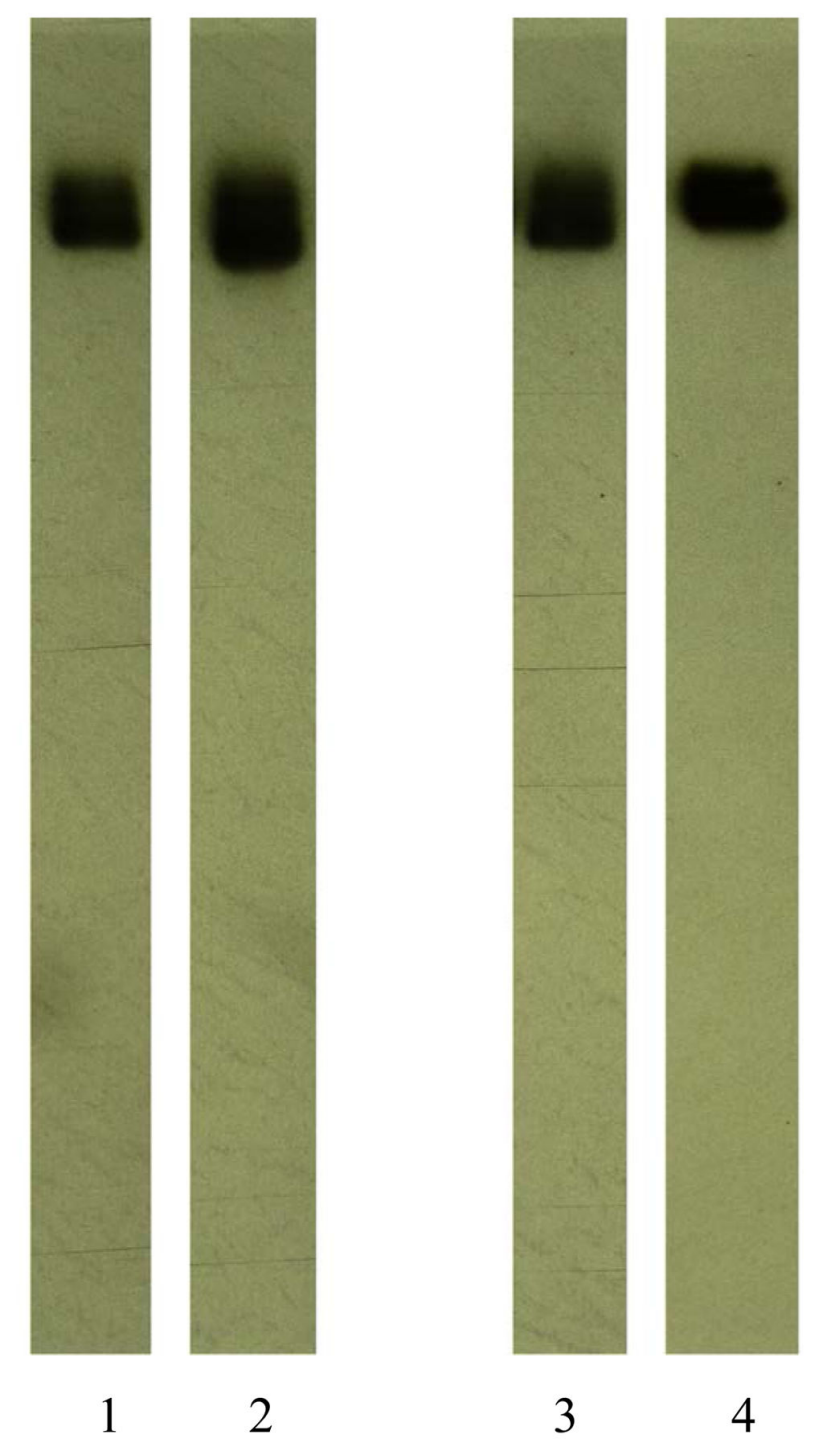
Characterization of the molecular weight profile for the covalent immunochemotherapeutics, gemcitabine-(C_4_-*amide*)-[anti-EGFR] and gemcitabine-(C_4_-*amide*)-[anti-HER2/*neu*] relative to reference control anti-EGFR and anti-HER2/*neu* monoclonal immunoglobulin fractions. *Legends*: (*Lane-*1) murine anti-human EGFR monoclonal immunoglobulin; (*Lane-*2) gemcitabine-(C_4_-*amide*)-[anti-EGFR]; (*Lane-*3) murine anti-human HER2/*neu* monoclonal immunoglobulin; and (*Lane-*4) gemcitabine-(C_4_-*amide*)-[anti-HER2/*neu*]. Covalent gemcitabine immunochemotherapeutics and monoclonal immunoglobulin fractions were size-separated by non-reducing SDS-PAGE followed by lateral transfer onto sheets of nitrocellulose membrane to facilitate detection with biotinylated goat anti-mouse IgG immunoglobulin. Subsequent analysis entailed incubation of membranes with strepavidin-HRPO in combination with the use of a HRPO chemiluminescent substrate for acquisition of autoradiography images.

Mass/size separation of covalent gemcitabine-(C_4_-*amide*)-[anti-HER2/*neu*] and gemcitabine-(C_4_-*amide*)-[anti-EGFR] immunochemotherapeutics by SDS-PAGE in combination with immunodetection analysis (Western blot) and chemiluminescent autoradiography recognized a single primary condensed band of 150-kDa between a molecular weight range of 5.0-kDa to 450-kDa (Figure 2). Patterns of low-molecular-weight fragmentation (proteolytic/hydrolytic degradation) or large-molecular-weight immunoglobulin polymerization were not detected (Figure 2). The observed molecular weight of 150-kDa for both gemcitabine-(C_4_-*amide*)-[anti-HER2/*neu*] and gemcitabine-(C_4_-*amide*)-[anti-EGFR] directly corresponds with the known molecular weight/mass of reference control anti-HER2/*neu* monoclonal immunoglobulin fractions (Figure 2). Analogous results have been reported for similar covalent immunochemotherapeutics [16] [18] [19] [24] [25] [27] [28].

#### Cell-Binding Analysis

**Figure 3:**
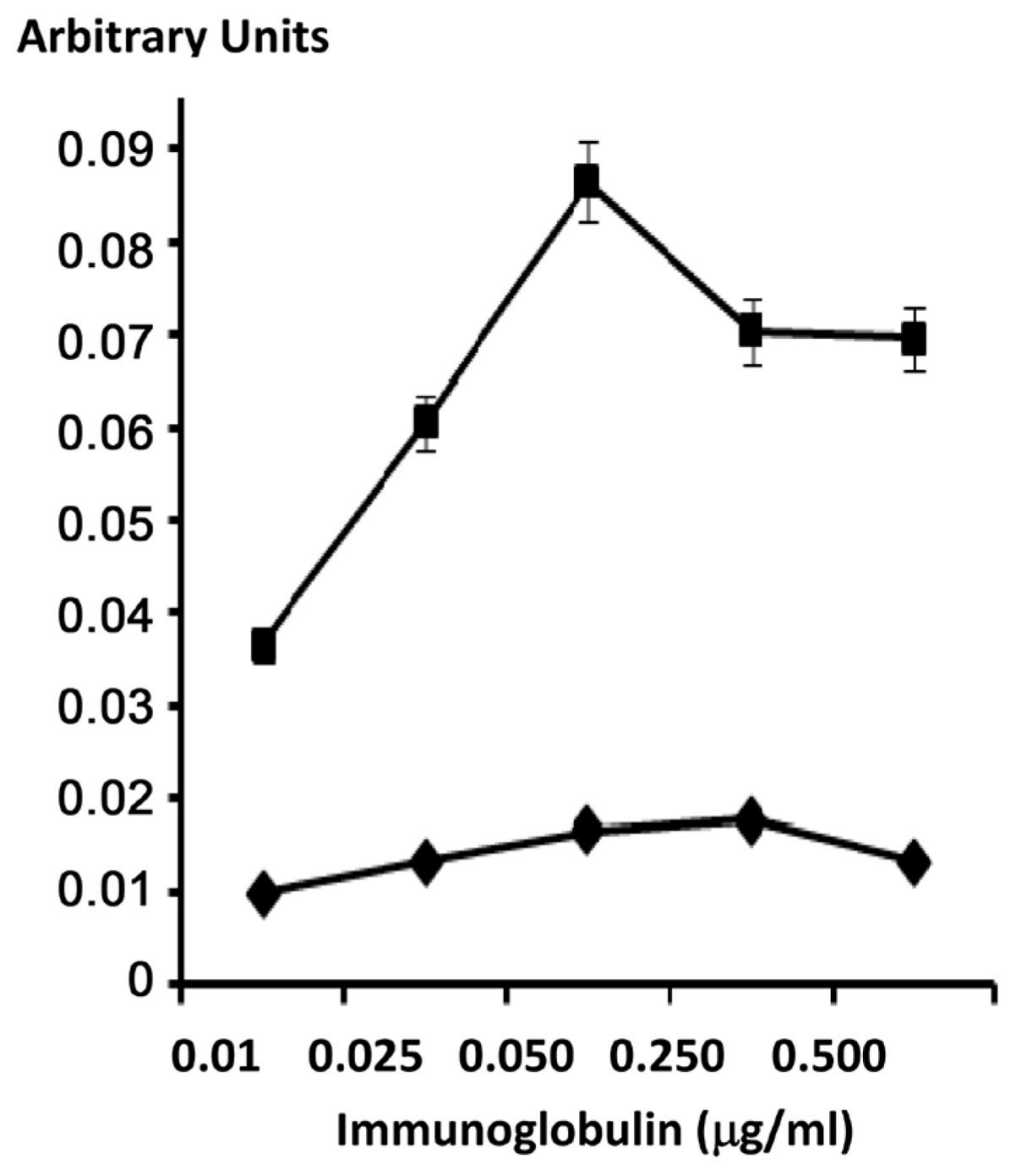
Detection of total immunoglobulin in the form of gemcitabine-(C_4_-*amide*)-[anti-EGFR] or gemcitabine-(C_4_-*amide*)-[anti-HER2/*neu*] selectively bound to the exterior surface membrane of mammary adenocarcinoma. *Legends*: (◆) gemcitabine-(C_4_-*amide*)-[anti-EGFR], and (■) gemcitabine-(C_4_-*amide*)-[anti-HER2/*neu*]. Covalent gemcitabine-(C_4_-*amide*)-[anti-EGFR] or gemcitabine-(C_4_-*amide*)-[anti-HER2/*neu*] immunochemotherapeutic formulated at gradient gemcitabine-equivalent concentrations were incubated in direct contact with triplicate monolayer populations of chemotherapeutic-resistant human mammary adenocarcinoma (SKBr-3) over a 4-hour time period. Total immunoglobulin bound to the exterior surface membrane was then detected and measured by cell-ELISA.

Total bound immunoglobulin in the form of gemcitabine-(C_4_-*amide*)-[anti-HER2/*neu*] or gemcitabine-(C_4_-*amide*)-[anti-EGFR] on the external surface membrane of adherent mammary adenocarcinoma (SKBr-3) populations was measured by cell-ELISA (Figure 3). Greater total membrane-bound gemcitabine-(C_4_-*amide*)-[anti-HER2/*neu*] was detected with progressive increases in standardized total immunoglobulin-equivalent concentrations formulated at 0.010, 0.025, 0.050, 0.250, and 0.500 μg/ml (Figure 3). In order to detect elevations in total membrane-bound gemcitabine-(C_4_-*amide*)-[anti-HER2/*neu*] or gemcitabine-(C_4_-*amide*)-[anti-EGFR] standardized total immunoglobulin-equivalent concentrations had to alternatively be formulated at 0.5, 1.0, 5.0 and 10.0 μg/ml (Figure 3). Collectively these results for the cell-ELISA analyses serve to validate the retained selective binding-avidity of gemcitabine-(C_4_-*amide*)-[anti-HER2/*neu*] and gemcitabine-(C_4_-*amide*)-[anti-EGFR] for external membrane HER2/*neu* receptor sites highly over-expressed at 1 × 10^6^/cell on the exterior surface membrane of mammary adenocarcinoma (SKBr-3) populations (Figure 3) [24].

#### Anti-neoplastic Cytotoxic Potency

**Figure 4:**
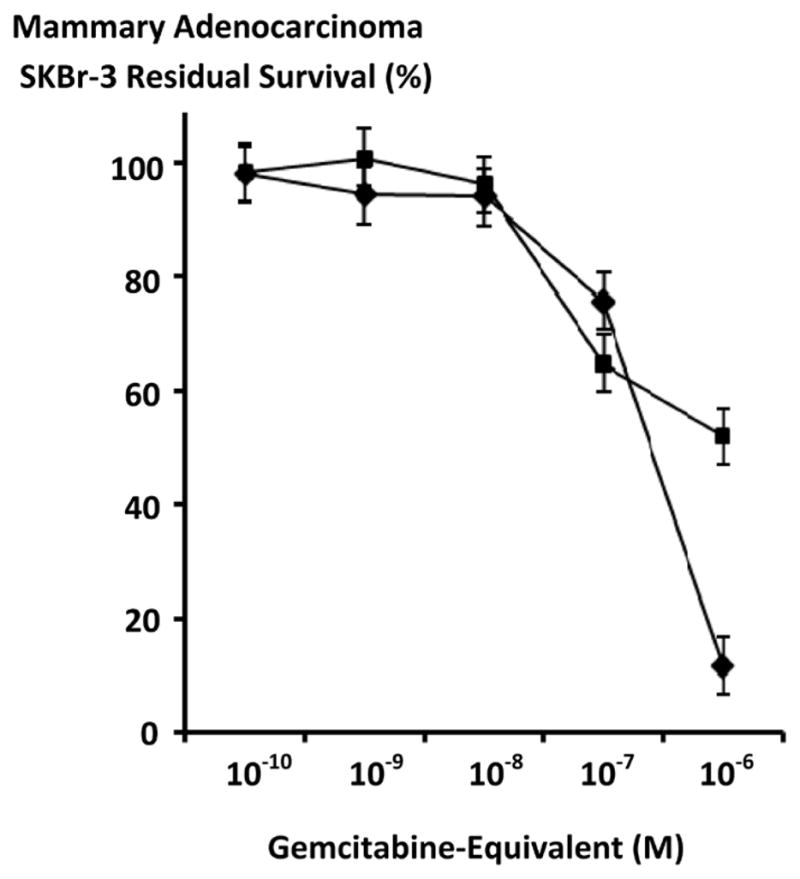
Relative gemcitabine anti-neoplastic cytotoxicity against chemotherapeutic-resistant mammary adenocarcinoma over challenge (incubation) periods of different duration. *Legends*: (■) gemcitabine following a 96-hour incubation period; and (◆) gemcitabine following a 182-hour incubation period. Gemcitabine formulated at gradient gemcitabine-equivalent concentrations was incubated in direct contact with triplicate monolayer populations of chemotherapeutic-resistant mammary adenocarcinoma (SKBr-3) for a period of either 96-hours or 182-hours. Anti-neoplastic cytotoxicity was measured using a MTT cell vitality assay relative to matched negative reference controls.

**Figure 5:**
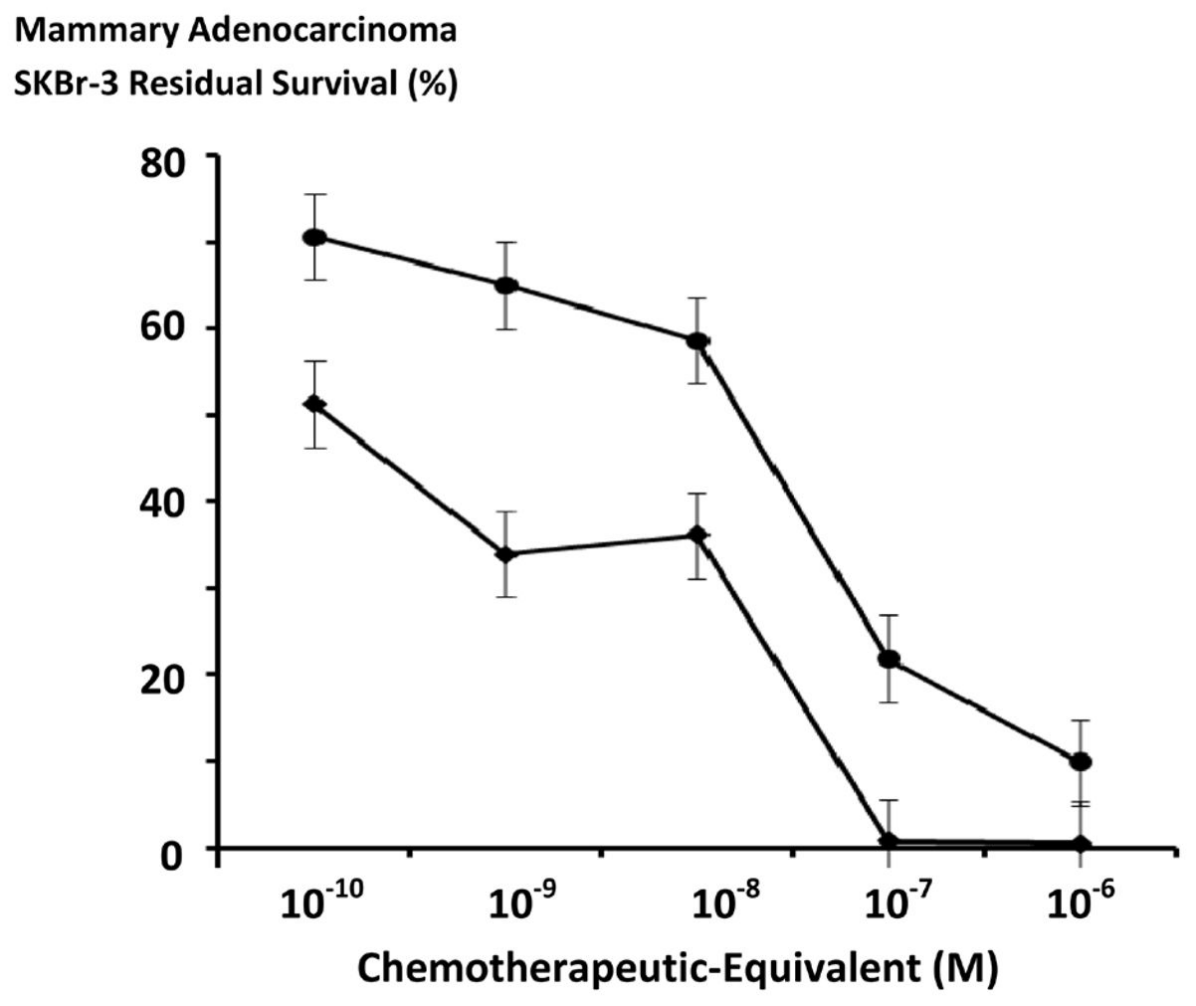
Relative anti-neoplastic cytotoxicity of gemcitabine-(C_4_-*amide*)-[anti-EGFR] against chemotherapeutic-resistant human mammary adenocarcinoma as a function of challenge (incubation) period duration. *Legends*: (■) gemcitabine-(C_4_-*amide*)-[anti-EGFR] following a 96-hour incubation period; and (◆) gemcitabine-(C_4_-*amide*)-[anti-EGFR] following a 182-hour incubation period. Covalent gemcitabine immunochemotherapeutic formulated at gradient gemcitabine-equivalent concentrations was incubated in direct contact with in triplicate monolayer populations of chemotherapeutic-resistant human mammary adenocarcinoma (SKBr-3) for a period of either 96-hours or 182-hours. Anti-neoplastic cytotoxicity was measured using an MTT cell vitality assay relative to matched negative reference controls.

**Figure 6:**
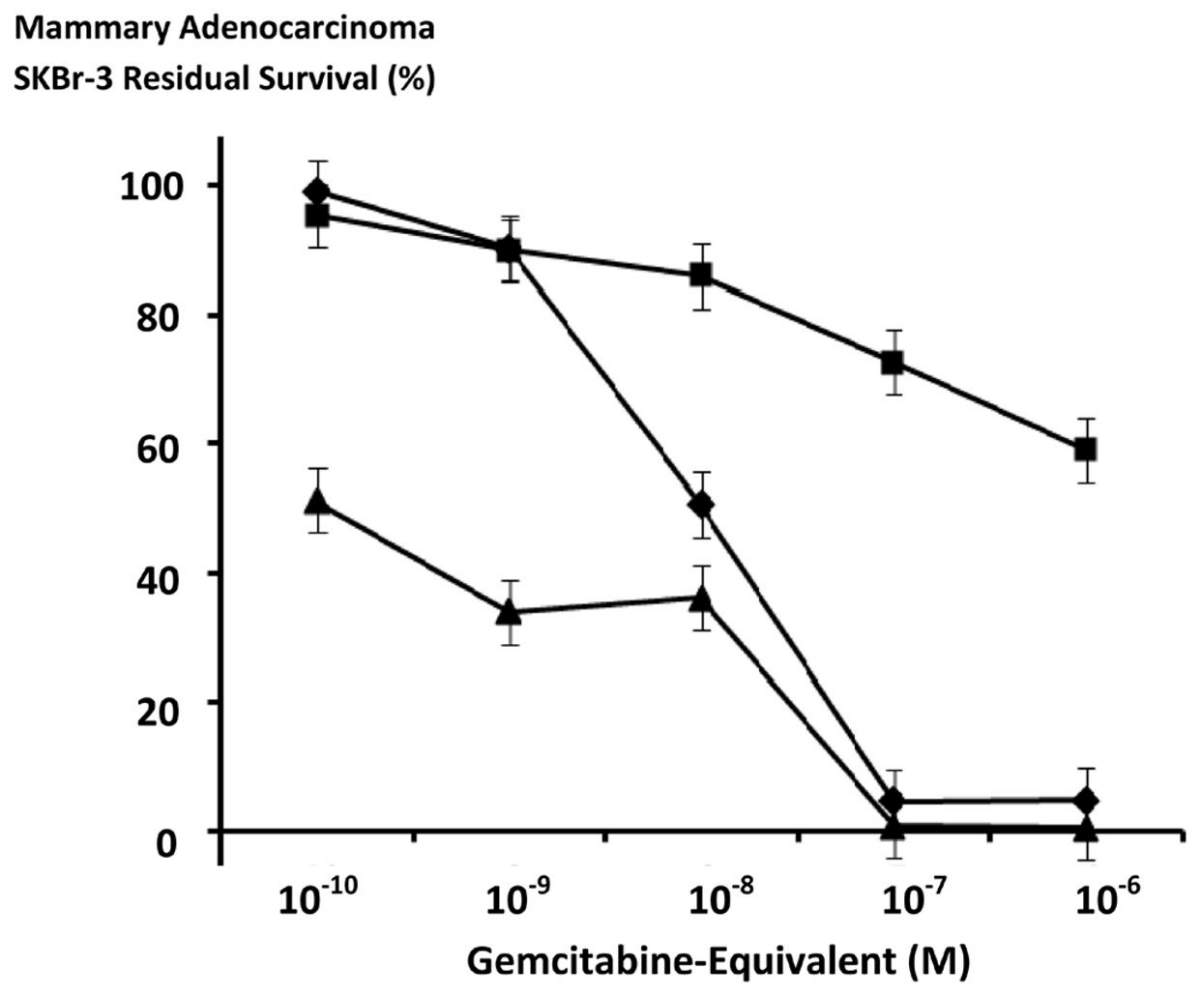
Relative anti-neoplastic cytotoxicity of covalent gemcitabine immunochemotherapeutics against chemotherapeutic-resistant human mammary adenocarcinoma. *Legends*: (▲) gemcitabine- (C_4_-*amide*)-[anti-EGFR] (182-hour incubation period); (■) gemcitabine-(C_4_-*amide*)-[anti-HER2/neu] (182-hour incubation period); and (◆) gemcitabine chemotherapeutic (96-hour incubation period). Chemotherapeutic-resistant mammary adenocarcinoma (SKBr-3) monolayer populations were incubated in direct contact with gemcitabine-(C_4_-*amide*)-[anti-EGFR], gemcitabine-(C_4_-*amide*)-[anti-HER2/*neu*], or gemcitabine formulated in triplicate at gradient gemcitabine-equivalent concentrations. Anti-neoplastic cytotoxicity was measured using a MTT cell vitality assay relative to matched negative reference controls.

Gemcitabine chemotherapeutic produced higher levels of anti-neoplastic cytotoxicity against chemotherapeutic resistant mammary adenocarcinoma during direct contact incubation periods of 182-hours compared to 96-hours especially at the gemcitabine-equivalent concentration of 10^−6^ M (Figure 4). Similarly, gemcitabine-(C_4_-*amide*)-[anti-EGFR] produced measurably higher levels of canti-neoplastic cytotoxicity when incubated with chemotherapeutic-resistant mammary adenocarcinoma (SKBr-3) for direct contact periods of 182-hours compared to 96-hours (Figure 5 and Figure 6).

**Figure 7:**
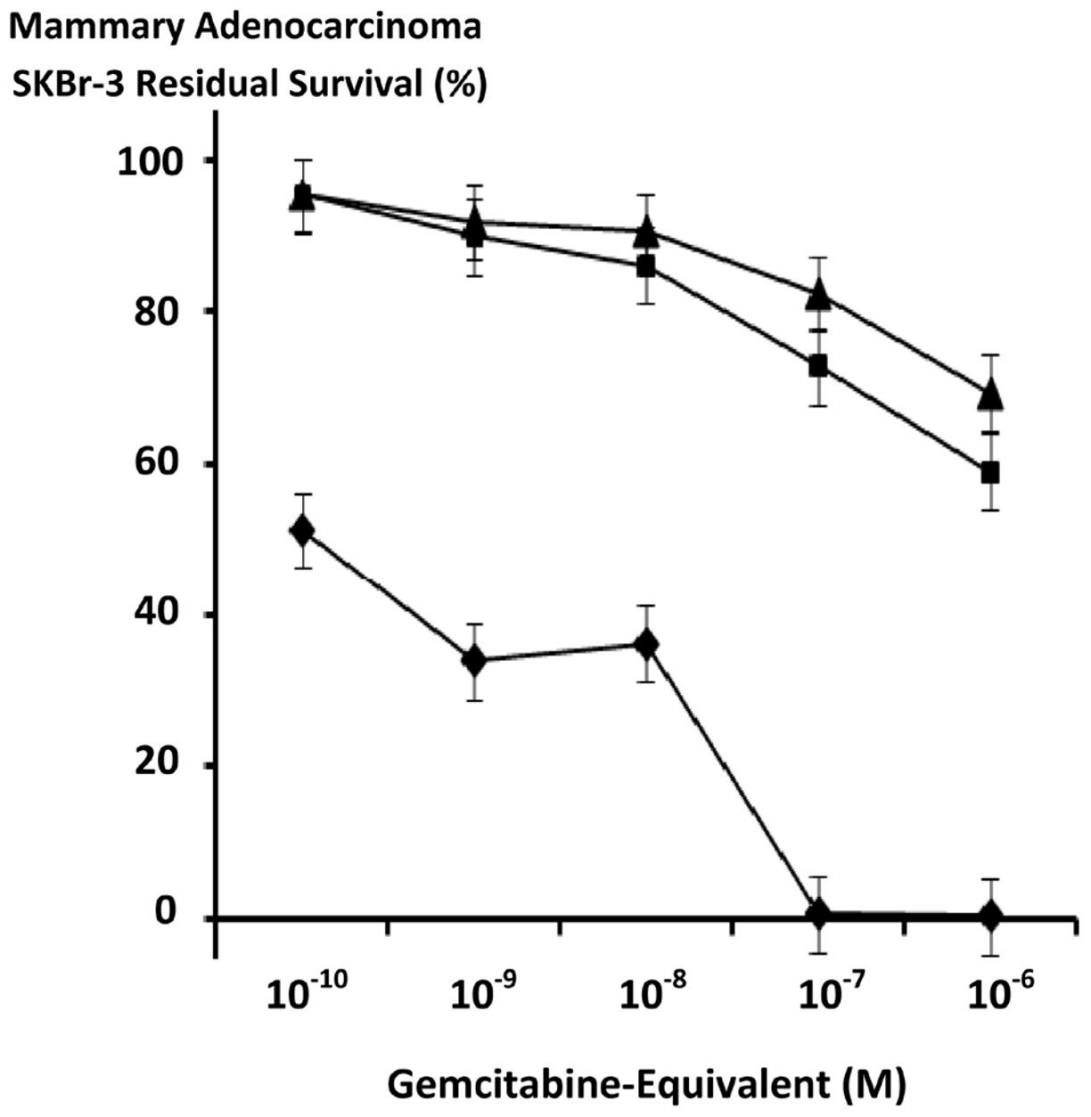
Relative anti-neoplastic cytotoxicity of three different covalent gemcitabine immunochemotherapeutics against chemotherapeutic-resistant human mammary adenocarcinoma. *Legends*: (◆) gemcitabine-(C_4_-*amide*)-[anti-EGFR]; (■) gemcitabine-(C_4_-*amide*)-[anti-HER2/*neu*] and (▲) gemcitabine-(C_5_-methylcarbamate)-[anti-HER2/*neu*]. Individual covalent gemcitabine immunochemotherapeutics formulated at gradient gemcitabine-equivalent concentrations were incubated in direct contact with triplicate monolayer populations of chemotherapeutic-resistant mammary adenocarcinoma (SKBr-3) for 182-hours. Anti-neoplastic cytotoxicity was measured using a MTT cell vitality assay relative to matched negative reference controls.

Anti-neoplastic cytotoxicity of gemcitabine-(C_4_-*amide*)-[anti-EGFR] against chemotherapeutic-resistant mammary adenocarcinoma (SKBr-3) was consistently greater with incubation periods of 182-hours compared to 96-hours (Figure 6 and Figure 7). Gemcitabine-(C_4_-*amide*)-[anti-EGFR] produced progressive anti-neoplastic cytotoxicity that increased with elevations in covalent immunochemotherapeutic at and between the gemcitabine-equivalent concentrations of 10^−10^ M and 10^−6^ M for incubation periods of 96-hours and 182-hours respectively (Figure 6 and Figure 7). The most rapid increases in anti-neoplastic cytotoxicity from 10.2% and 48.7% (89.8% and 51.3% residual survival) to 95.3% and 99.3% (4.7% and 0.7% residual survival) were detected at and between the gemcitabine-equivalent concentrations of 10^−9^ M-to-10^−7^ M and 10^−10^ M-to-10^−7^ M at 96 hours and 182-hours respectively (Figure 6 and Figure 7). Maximum but only slight higher anti-neoplastic cytotoxicity of 95.3% and 99.6% (4.7% and 0.4% residual survival) was detected with gemcitabine-(C_4_-*amide*)-[anti-EGFR] at 10^−6^ M for incubation periods of 96-hours and 182-hours respectively (Figure 6 and Figure 7).

Gemcitabine-(C_4_-*amide*)-[anti-EGFR] produced greater anti-neoplastic cytotoxicity than either gemcitabine (C_5_-*methylcarbamate*)-[anti-HER2/*neu*] [24] or gemcitabine-(C_4_-*amide*)-[anti-HER2/*neu*] against chemotherapeutic-resistant mammary adenocarcinoma (SKBr-3) during an 182-hour incubation period (Figure 6 and Figure 7). Gemcitabine-(C_4_-*amide*)-[anti-HER2/*neu*] and gemcitabine (C_5_-*methylcarbamate*)-[anti-HER2/*neu*] anti-neoplastic cytotoxicity levels of 14.4% and 9.6% (85.9% and 90.4% residual survival) compared to 63.9% (36.1% residual survival) for gemcitabine-(C_4_-*amide*)-[anti-EGFR] at the gemcitabine-equivalent concentration of 10^−8^ M (Figure 6 and Figure 7). Gemcitabine-(C_4_-*amide*)-[anti-HER2/*neu*] and gemcitabine (C_5_-*methylcarbamate*)-[anti-HER2/*neu*] both produced progressive but modest increases in anti-neoplastic cytotoxicity that most rapidly increased at and between 10^−8^ M (85.9% and 90.4% residual survival) and 10^−6^ M (58.9% and 69.2% residual survival). In contrast, gemcitabine-(C_4_-*amide*)-[anti-EGFR] produced anti-neoplastic cytotoxicity levels of 63.9%, 99.3% and 99.6% (36.1%, 0.7%, 0.4% residual survival) at gemcitabine-equivalent concentrations of 10^−8^ M, 10^−7^ M and 10^−6^ M respectively (Figure 6 and Figure 7). Maximum cytotoxic anti-neoplastic potencies for gemcitabine-(C_4_-*amide*)-[anti-EGFR], gemcitabine-(C_4_-*amide*)-[anti-HER2/*neu*] and gemcitabine (C_5_-*methylcarbamate*)-[anti-HER2/*neu*] were 99.6%, 41.1% and 30.8% (0.4%, 59.0% and 69.2% residual survival) at the gemcitabine-equivalent concentration of 10^−6^ M respectively (Figure 6 and Figure 7).

Gemcitabine-(C_4_-*amide*)-[anti-EGFR] compared to gemcitabine alone produced higher levels of anti-neoplastic cytotoxicity against chemotherapeutic-resistant mammary adenocarcinoma (SKBr-3) at the gemcitabine- equivalent concentrations of 10^−10^ M (51.3%-vs-99.0% residual survival), 10^−9^ M (34.0%-vs-90.2% residual survival), and 10^−8^ M (36.1%-vs-50.6% residual survival) respectively (Figure 6) Nearly identical maximum anti-neoplastic cytotoxicity levels of 99.3%-vs-95.3% (0.7% and 4.7% residual survival) and 99.6%-vs-95.3% (0.4% and 4.7% residual survival) were detected for gemcitabine-(C_4_-*amide*)-[anti-EGFR] and gemcitabine at the gemcitabine-equivalent concentrations of 10^−7^ M and 10^−6^ M respectively (Figure 6). Gemcitabine-(C_4_-*amide*)-[anti-HER2/*neu*] and gemcitabine had nearly identical anti-neoplastic cytotoxicity against chemotherapeutic-resistant mammary adenocarcinoma (SKBr-3) at gemcitabine-equivalent concentrations of 10^−10^ M and 10^−9^ M but gemcitabine was much more potent at 10^−8^ M (85.9%-vs-50.6% residual survival), 10^−9^ M (4.6%-vs-72.7% residual survival), and 10^−6^ M (4.7%-vs-58.9% residual survival) respectively (Figure 6).

**Figure 8:**
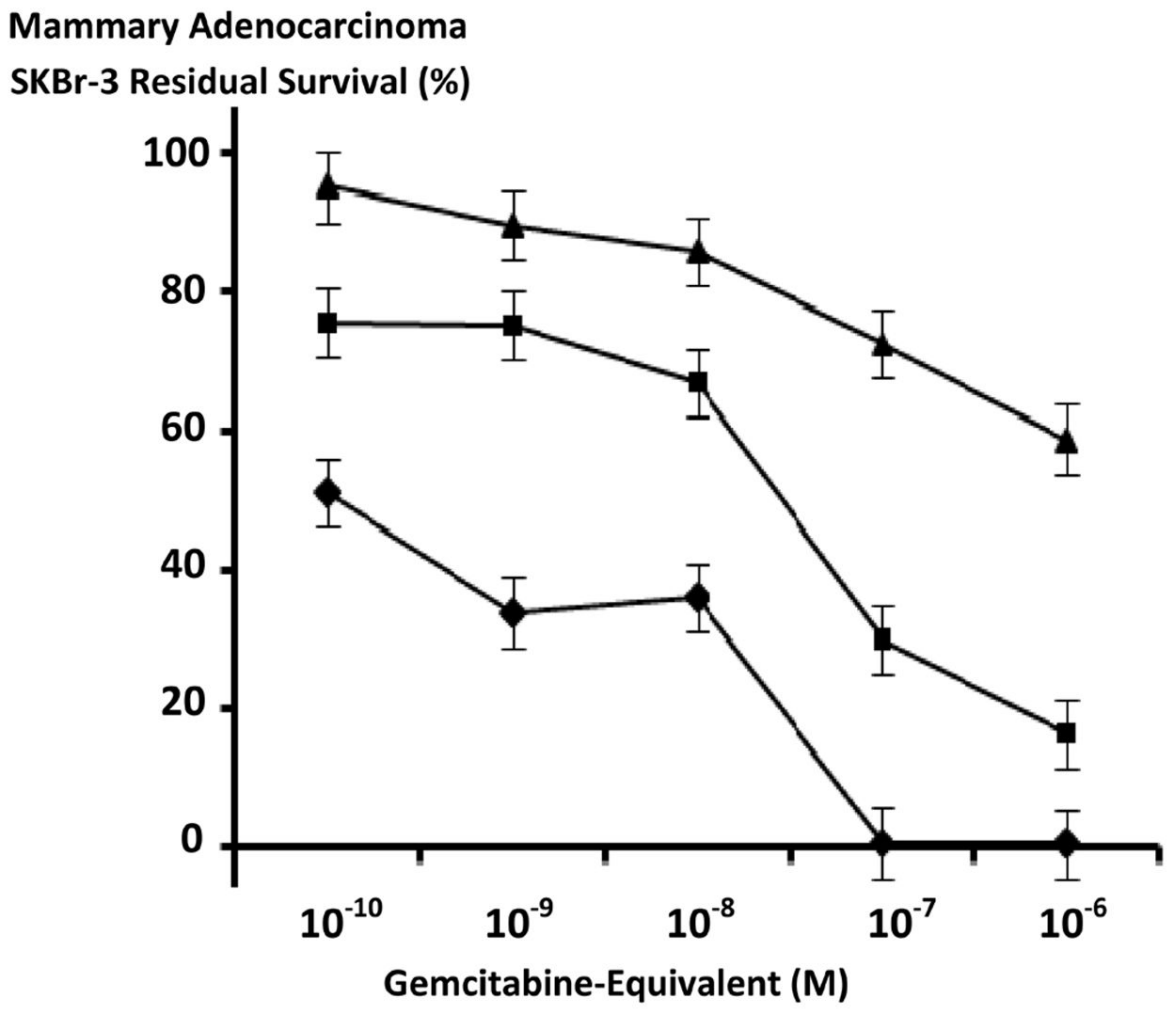
Relative anti-neoplastic cytotoxicity of individual and dual simultaneous combinations of covalent gemcitabine immunochemotherapeutics against chemotherapeutic-resistant human mammary adenocarcinoma. *Legends*: (◆) gemcitabine-(C_4_-*amide*)-[anti-EGFR]; (▲) gemcitabine-(C_4_-*amide*)-[anti-HER2/*neu*]; and (■) gemcitabine-(C_4_-*amide*)-[anti-EGFR] with gemcitabine-(C_4_-*amide*)-[anti-HER2/*neu*]. Individual or dual simultaneous combinations of covalent gemcitabine immunochemotherapeutics formulated at gradient 50/50 gemcitabine-equivalent concentrations were incubated in direct contact with triplicate monolayer populations of chemotherapeutic-resistant mammary adenocarcinoma (SKBr-3) for a period of 182-hours. Anti-neoplastic cytotoxicity was measured using a MTT cell vitality assay relative to matched negative reference controls.

The dual simultaneous combination of gemcitabine-(C_4_-*amide*)-[anti-EGFR] with gemcitabine-(C_4_-*amide*)-[anti-HER2/*neu*] evaluated as a gemcitabine-standardized 50/50 molar equivalent formulation in addition to gemcitabine-(C_4_-*amide*)-[anti-EGFR] and gemcitabine-(C_4_-*amide*)-[anti-HER2/*neu*] all produced progressive increases in anti-neoplastic cytotoxicity against chemotherapeutic resistant mammary adenocarcinoma (SKBr-3) as a function of increases in gemcitabine-equivalent concentration at and between 10^−10^ M and 10^−6^ M (Figure 8). Anti-neoplastic cytotoxicity of gemcitabine-(C_4_-*amide*)-[anti-EGFR] in dual simultaneous combination with gemcitabine-(C_4_-*amide*)-[anti-HER2/*neu*] was 24.3%, 24.5%, 32.8%, 69.9%. and 83.7% (75.7%, 75.5%, 67.2%, 30.1% and 16.3% residual survival) at the gemcitabine-equivalent concentrations of 10^−10^ M, 10^−9^ M, 10^−8^ M, 10^−7^ M and 10^−6^ M respectively (Figure 8). Gemcitabine-(C_4_-*amide*)-[anti-EGFR] in dual simultaneous combination with gemcitabine-(C_4_-*amide*)-[anti-HER2/*neu*] produced levels of anti-neoplastic cytotoxicity that were less than for gemcitabine-(C_4_-*amide*)-[anti-EGFR] but consistently greater than levels for gemcitabine-(C_4_-*amide*)-[anti-HER2/*neu*] at and between the gemcitabine-equivalent concentrations of 10^−10^ M and 10^−6^ M respectively (Figure 8). Anti-neoplastic cytotoxicity for gemcitabine-(C_4_-*amide*)-[anti-EGFR] in dual simultaneous combination with gemcitabine-(C_4_-*amide*)-[anti-HER2/*neu*] compared to gemcitabine-(C_4_-*amide*)-[anti-EGFR] and gemcitabine-(C_4_-*amide*)-[anti-HER2/*neu*] reached maximum or near maximum levels of 83.7%, 99.6%, and 30.8% (16.3%, 0.4%, and 69.2% residual survival) respectively (Figure 8).

**Figure 9:**
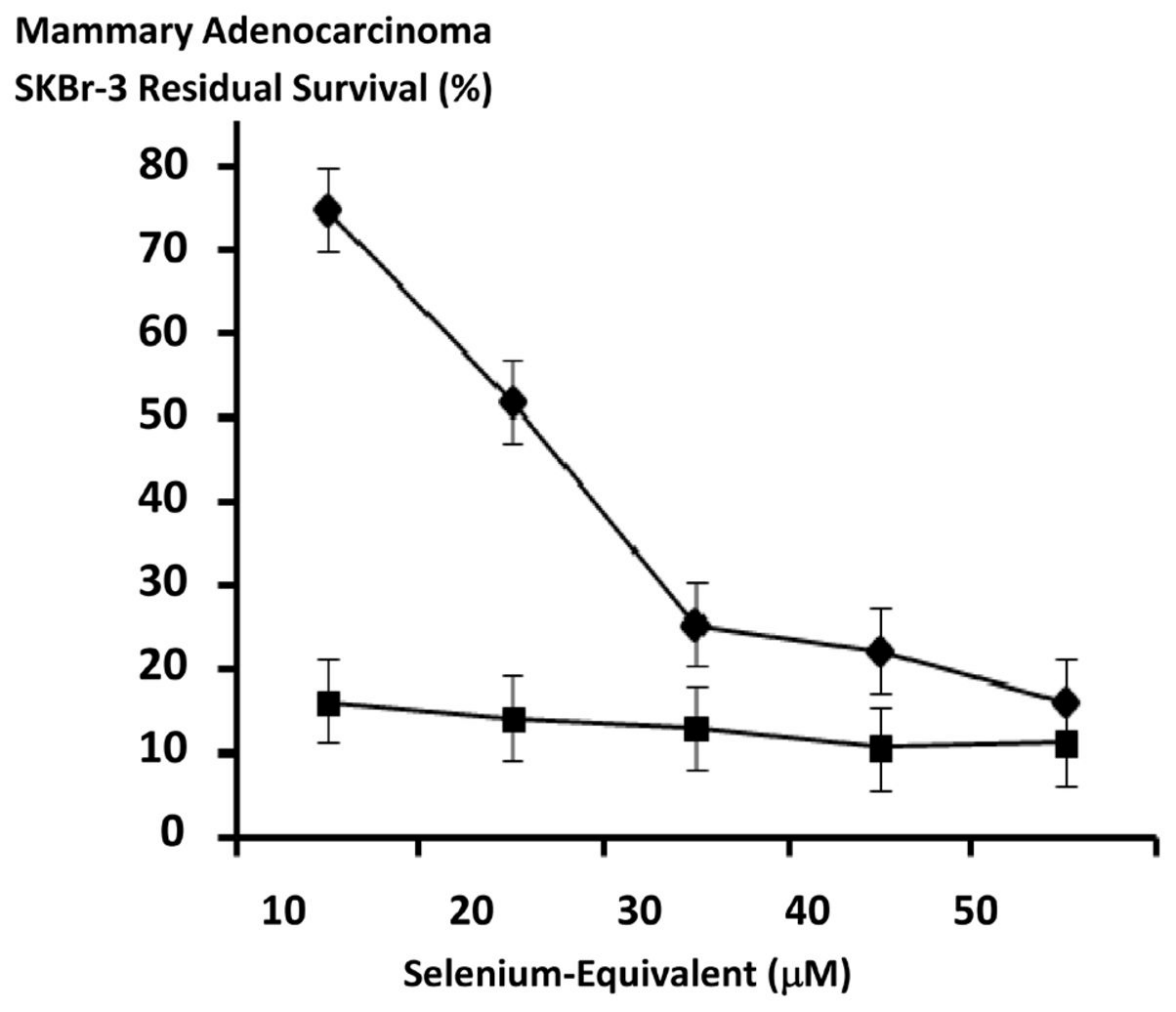
Relative anti-neoplastic cytotoxicity of organoselenium compounds against chemotherapeutic-resistant human mammary adenocarcinoma. *Legends*: (◆) [Se]-methylselenocysteine; and (■) methylseleninate. Individual organoselenium compounds formulated at gradient selenium-equivalent concentrations was incubated in direct contact with triplicate populations of chemotherapeutic-resistant mammary adenocarcinoma (SKBr-3). Anti-neoplastic cytotoxicity was measured using a MTT cell vitality assay relative to matched negative reference controls.

**Figure 10:**
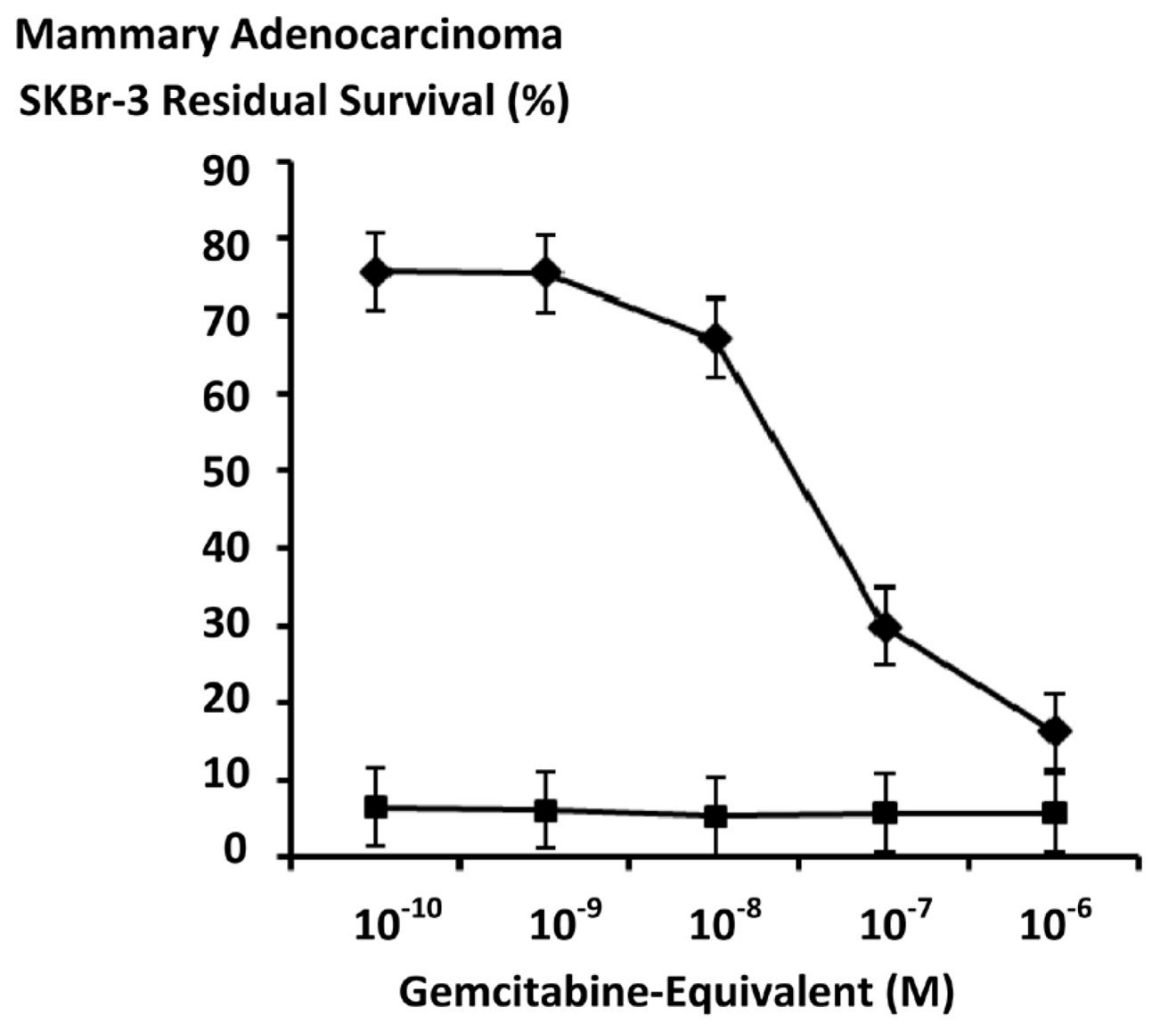
Relative anti-neoplastic cytotoxicity for dual simultaneous combinations of two different covalent gemcitabine-immunochemotherapeutics enhanced by [Se]-methylselenocysteine against chemotherapeutic-resistant human mammary adenocarcinoma. *Legends*: (◆) gemcitabine-(C_4_-*amide*)-[anti-EGFR] with gemcitabine-(C_4_-*amide*)-[anti-HER2/*neu*]; and (■) gemcitabine-(C_4_-*amide*)-[anti-EGFR] with gemcitabine-(C_4_-*amide*)-[anti-HER2/*neu*] in the presence of a fixed concentration of [Se]-methyl- cysteine (15 μM). The dual simultaneous combination of covalent gemcitabine-immunochemotherapeutics (+/− [Se]-methylcysteine) was formulated at gradient 50/50 gemcitabine-equivalent concentrations and incubated in direct contact for **96-hours** with triplicate monolayer populations of chemotherapeutic-resistant human mammary adenocarcinoma (SKBr-3). Anti-neoplastic cytotoxicity was measured using a MTT cell vitality assay relative to matched negative reference controls.

Methylseleninate produced levels of anti-neoplastic cytotoxicity that were substantially greater than those detected for [Se]-methylselenocysteine at the selenium-equivalent concentrations of 10 μM, and 20 μM but approached similar levels at 30 μM and 40 μM with essentially equivalent potency observed at 50 mM respectively (Figure 9). Methylseninate had almost equivalent maximal levels of anti-neoplastic cytotoxicity at and between the selenium-equivalent concentrations of 10 μM and 50 μM while [Se]-methylselenocysteine produced rapid progressive increases in anti-neoplastic cytotoxicity at and between 10 μM and 30 μM while levels were near maximum at 30 μM, 20 μM and 10 μM (Figure 9). [Se]-methylselenocysteine substantially contributed to the anti-neoplastic cytotoxicity of gemcitabine-standardized 50/50 formulations of gemcitabine-(C_4_-*amide*)-[anti-EGFR] with gemcitabine-(C_4_-*amide*)-[anti-HER2/*neu*] compared to *only* the dual simultaneous combination of the two covalent gemcitabine immunochemotherapeutics (Figure 8 and Figure 10). Gemcitabine-(C_4_-*amide*)-[anti-EGFR] and gemcitabine-(C_4_-*amide*)-[anti-HER2/*neu*] produced progressive increases in anti-neoplastic cytotoxicity that were most rapid at and between the gemcitabine-equivalent concentrations of 10^−9^ M and 10^−6^ M (Figure 8 and Figure 10). [Se]-methylselenocysteine (15 μM final concentration) in combination with the two covalent gemcitabine immunochemotherapeutics resulted in anti-neoplastic cytotoxicity levels of 93.5% at 10^−10^ M (6.5% residual survival), 93.9% at 10^−9^ M (6.1% residual survival), 94.7% at 10^−8^ M (5.3% residual survival), 94.2% at 10^−7^ M (5.8% residual survival), and 94.2% at 10^−6^ M (5.8% residual survival) following a direct-contact incubation period (Figure 10).

## 4. Discussion

Despite their common application in modern clinical oncology, conventional chemotherapeutics when given as a monotherapy at dosages that produce safe plasma concentrations almost invariably lack sufficient potency or efficacy to completely resolve most neoplastic disease states. Even when applied as prescribed chemotherapeutic administration is frequently accompanied by some degree of risk for inducing serious sequelae especially during periods of long-term utilization. Newer treatment modalities such as monoclonal immunoglobulin that inhibit function of trophic membrane receptors frequently over-expressed by many neoplastic cell types offer an opportunity to avoid many of the common side effects associated with conventional chemotherapeutics. Unfortunately, most monoclonal immunoglobulin-based therapies that inhibit function of HER2/*neu*, EGFR, VEGF, IGFR and other uniquely or highly over-expressed trophic receptors are usually only capable of promoting declines in proliferation rate and are largely incapable of evoking cytotoxic activity sufficient to effectively resolve most aggressive or advanced forms of neoplastic disease [7]–[12] [29]–[39]. Inability of most anti-trophic immunoglobulins to exert significant cytotoxic efficacy *in-vivo* is in part associated with the detection of increases in cell-cycle G_1_-arrest, cellular transformation to states of apoptosis-resistance [30], and selection for resistant sub-populations [31] [35] that can be further complicated by frequent reversal of tumor growth inhibition [31] and resumed trophic receptor over-expression [29] following discontinuation of immunoglobulin therapy. Greater levels of anti-neoplastic cytotoxicity are alternatively attainable when anti-trophic receptor immunoglobulin are utilized in dual combination with conventional chemotherapeutics or other cancer treatment modalities [13]–[15].

A small collection of semi-synthetic heterobifunctional organic chemistry reactions can be used to covalently bond gemcitabine to monoclonal immunoglobulin, receptor ligands (e.g. EGFR) or other biologically active protein fractions. One potential method involves creation of a covalent bond structure at the cytosine 2 monoamine group of gemcitabine [40]–[44] either as a direct covalent bond to a ligand or for the purpose of creating a chemically reactive gemcitabine intermediate. Similar molecular strategies have been employed to synthesize covalent anthracycline immunochemotherapeutics through the creation of a covalent bond structure at the *α*-monoamine (C_3_-*amino*) group of the carbohydrate-like moiety of doxorubicin, daunorubicin, epirubicin and other related agents in this class of chemotherapeutics [16] [45]–[56]. Generation of a covalent bond at the C_5_-*methylhydroxy* group of gemcitabine represents an alternative molecular strategy for the synthesis covalent gemcitabine-ligand biopharmaceuticals [41] [44] [57]–[61].

Gemcitabine has been covalent bonded to a number of biologically relevant ligands. Most prominent in this regard has been poly-L-glutamic acid (polypeptide configuration) [60]; cardiolipin [57] [58]; 1-dodecylthio-2-decyloxypropyl-3-phophatidic acid [59] [61]; lipid-nucleosides [62]; *N*-(2-hydroxypropyl)methacrylamide polymer (HPMA) [40]; benzodiazepine receptor ligand [41] [44]; 4-(*N*)-valeroyl, 4-(*N*)-lauroyl, 4-(*N*)-stearoyl [43], and anti-HER2/neu [19] [24]; in addition to 4-fluoro[18F]-benzaldehyde derivative [42] for application as a diagnostic positron emitting radionuclide. Few if any reports have described the molecular design, synthesis and efficacy evaluation of a covalent gemcitabine immunochemotherapeutic produced through the generation of co-valent bond structures at either the cytosine-like C_4_ mono-*amine* [19] or C_5_-*methylhydroxy* groups [24].

Covalent immunochemotherapeutics can be synthesized that promote selective “targeted” chemotherapeutic delivery in a manner that evoke greater levels of anti-neoplastic cytotoxic potency than the corresponding non-covalent “*free*” or “*parent*” form of a chemotherapeutic moiety [16] [18] [25] [63]–[68]. Several molecular mechanisms and cellular processes can be modulated for the purpose of optimizing and enhancing properties that ultimately influence anti-neoplastic cytotoxic potency. Biological activity of the immunoglobulin component of gemcitabine-(C_4_-*amide*)-[anti-EGFR] and gemcitabine-(C_4_-*amide*)-[anti-HER2/*neu*] directly facilitates their binding-avidity for trophic membrane receptor sites (e.g. anti-EGFR, anti-HER2/*neu*) that in turn affords several properties which significantly contribute to the total anti-neoplastic cytotoxic potency of these covalent immunochemotherapeutics. Monoclonal immunoglobulin selected for the synthesis of covalent immunochemotherapeutics should ideally possess several distinct properties that include selective binding-avidity for specific antigenic “sites” on the external surface membrane of cancer cells that are themselves uniquely or highly over-expressed compared to normal, healthy tissues and organ systems. Utilizing immunoglobulin fractions that possess these characteristics allows them to effectively function as a molecular platform that can facilitate selective “targeted” chemotherapeutic delivery in addition to the potential capacity to promote progressive and continual membrane deposition of the chemotherapeutic moiety. The chemotherapeutic-resistant mammary adenocarcinoma (SKBr-3) cell type over-expresses EGFR (2.2 × 10^5^/cell) and highly over-expresses HER2/*neu* (1 × 10^6^/cell) on its exterior surface membrane which promotes selectively “targeted” delivery and progressive membrane deposition of gemcitabine-(C_4_-*amide*)-[anti-EGFR] and gemcitabine-(C_4_-*amide*)-[anti-HER2/*neu*] at two different endogenous trophic membrane receptor sites. Progressive membrane deposition of gemcitabine-(C_4_-*amide*)-[anti-EGFR], gemcitabine-(C_4_-*amide*)-[anti-HER2/*neu*] or any other analogous covalent immunochemotherapeutic continues as long as sufficient covalent immunochemotherapeutic is present and EGFR and HER2/*neu* are expressed and re-expressed on the exterior surface membrane. Given this perspective, one of the most critically important mathematical variables related to cancer cell biology that can significantly determine the anti-neoplastic cytotoxicity of covalent immunochemotherapeutics like gemcitabine-[anti-HER2/*neu*], [19] [24] gemcitabine-[anti-EGFR], epirubicin-[anti-HER2/*neu*] [16] [18] [25] or epirubicin-[anti-EGFR], [16] is the expression density of “sites” on the external surface membrane of neoplastic cells utilized to facilitate the selective “targeted” delivery of chemotherapeutic moieties.

In direct accord with the inter-dependent relationship between the immunoglobulin component of covalent immunochemotherapeutics and the biological characteristics of neoplastic cell types, there are other variables in addition to the expression density of membrane-associated “target” sites that significantly determine the anti-neoplastic cytotoxicity of gemcitabine-[anti-HER2/*neu*], [19] [24] gemcitabine-[anti-EGFR], epirubicin-[anti-HER2/*neu*], [16] [18] [25] epirubicin-[anti-EGFR], [16] and similar covalent immunochemotherapeutics. When uniquely or over-expressed endogenous receptors that are actively internalized by processes of receptor-mediated endocytosis [69] have been selected as sites to facilitate the selective “targeted” delivery and membrane deposition of a chemotherapeutic moieties, it then becomes possible to minimize or avoid simple “coating” of the exterior surface membrane with covalent immunochemotherapeutics like gemcitabine-(C_4_-*amide*)-[anti-EGFR] and gemcitabine-(C_4_-*amide*)-[anti-HER2/*neu*]. Importance of this consideration is based on the realization that in general, it is a prerequisite for most classical chemotherapeutic agents like gemcitabine that possess mechanisms-of-action that is dependent upon enter into the cytosol or nuclear environments in order to create a biological effect. Such processes are assumed to not be a requirement for anti-cancer therapeutics that are membrane-active agents or radioimmunopharmaceuticals that have mechanisms-of-action that do not require entry into cytosol or nuclear environments (e.g. [^213^Bi or ^211^At or ^224^Ra]-anti-TAG-72 for colon carcinoma).

Uniquely or over-expressed endogenous receptor types known to be actively internalized by mechanisms of receptor-mediated endocytosis in response to physical binding of immunoglobulin or receptor ligands represents one of the more preferred type of sites on exterior surface membrane of neoplastic populations that can be utilized to selectively “target” chemotherapeutic delivery while also potentially facilitating profound cytosol chemotherapeutic moiety accumulation [69] in addition to preventing or minimizing distribution into and deposition within populations of non-neoplastic cell types (e.g. normal tissues and healthy organ systems). Between different endogenous receptor types and different neoplastic cell populations, variations undoubtedly exist in the rate and extent to which covalent immunochemotherapeutics are deposited on the external surface membrane and are subsequently internalized following the initiation of receptor-mediated-endocytosis [69]. Although specific data for EGFR and HER2/*neu* receptor-mediated endocytosis in populations of mammary adenocarcinoma (SKBr-3) is somewhat limited, other neoplastic cell types like metastatic multiple myeloma are known to internalize and metabolize approximately 8 × 10^6^ molecules of anti-CD74 monoclonal antibody per day [70]. In this context, the collective implications of; [*i*] selective “targeted” delivery and physical binding at over-expressed and highly over-expressed endogenous receptor sites (e.g. EGFR, HER2/*neu*); [*ii*] continual and progressive membrane deposition; [*iii*] initiation of receptor-mediated endocytosis; and [*iv*] re-expression/replenishment of uniquely or highly over-expressed endogenous receptors is the potential for gemcitabine-(C_4_-*amide*)-[anti-EGFR] and gem-citabine-(C_4_-*amide*)-[anti-HER2/*neu*] and analogous covalent immunochemotherapeutics to promote chemothe-rapeutic moiety accumulation within the cytosol. The degree of cytosol accumulation can approach concentrations that are 8.5× [67] to 100× [71] fold greater than levels attainable by simple passive diffusion of most conventional small molecular weight chemotherapeutics from the extracellular fluid compartment following intravenous administration at clinically-relevant (safe) dosages. Intracellular accumulation of chemotherapeutic moieties of covalent immunochemotherapeutics can therefore continue to occur in neoplastic populations that have been sub-lethally injured as long as they retain the capacity to be uniquely or highly over-express which can be directly influence by the rate at which endogenous membrane receptors are replenished following initial phases of active internalization by mechanisms of receptor-mediated endocytosis [69]. The degree to which such phenomenon occur therefore directly influences and contributes to the potency of gemcitabine-(C_4_-*amide*)-[anti-EGFR], gemcitabine-(C_4_-*amide*)-[anti-HER2/*neu*], gemcitabine-EGF and analogous covalent biochemotherapeutics. Conservative speculation suggests that dual-combinations of covalent immunochemotherapeutics like gemcitabine-(C_4_-*amide*)-[anti-EGFR] with gemcitabine-(C_4_-*amide*)-[anti-HER2/*neu*] promote greater levels of simultaneous selective “targeted” gemcitabine delivery/membrane deposition and intracellular gemcitabine internalization at both EGFR and HER2/*neu* endogenous receptors than can be achieved through selective “targeted” gemcitabine delivery at only a single endogenous membrane receptor over-expressed on the exterior surface membrane of chemotherapeutic-resistant mammary adenocarcinoma (SKBr-3). The promotion of relatively high cytosol chemotherapeutic concentrations within a short confined time period at least in theory decreases the opportunity and frequency that neoplastic cell sub-populations can develop certain forms of (acquired) chemotherapeutic-resistance.

Enhanced levels of anti-neoplastic cytotoxicity that are potentially attainable with a dual-combination of gemcitabine-(C_4_-*amide*)-[anti-EGFR] and gemcitabine-(C_4_-*amide*)-[anti-HER2/*neu*] chemotherapeutic-resistant neoplastic cell types can be attributed to physical properties associated with the relatively large molecular weight of selective delivery platforms that chemotherapeutic moieties are often covalently bound to (e.g. IgG MW = 150,000 vs gemcitabine MW = 263.198). Covalent bonding of chemotherapeutics to molecular delivery platforms of relatively large molecular weight effectively prolongs the intravascular pharmacokinetic profile of chemotherapeutic moieties in part because they are no longer removed as rapidly or as extensively from the plasma compartment by renal glomerular filtration (MWCO = 50 – 60 kDa) and excreted into the urine. Furthermore, the chemotherapeutic moiety of covalent immunochemotherapeutic agents do not distribute as extensively into cell populations residing within normal tissues and healthy organ systems because of the large molecular weight of the selective delivery platform (e.g. IgG = 150-kDa) which prevents simple passive diffusion across intact lipid bilayer membranes. The latter consideration is significant because a significantly large percentage of the total dose for a conventional small molecular weight chemotherapeutics within the intravascular compartment ultimately does passively diffuse across and enter the cytosol environment of cell populations in normal tissues and healthy organ systems.

The large molecular weight of the immunoglobulin component of gemcitabine-(C_4_-*amide*)-[anti-EGFR] and gemcitabine-(C_4_-*amide*)-[anti-HER2/*neu*] or analogous covalent immunochemotherapeutics also inhibits through mechanisms of steric hinderance, the function of biological entities that can utilize chemotherapeutics moieties as a molecular substrate. Enzymes like cytosine deaminase are not as efficient in biochemically degrading or inactivate gemcitabine when it is a moiety within a covalent immunochemotherapeutic. Presumably, at least some degree steric hinderance phenomenon are also responsible for the observation that the non-selective transmembrane efflux “pump”, P-glycoprotein (MDR-1: multi-drug resistance protein) [59] commonly responsible for imparting chemotherapeutic-resistance in many neoplastic cell types [72]–[77] is less effective in promoting resistance when chemotherapeutic moieties are formulated as covalent immunochemotherapeutics [51] [77]–[80]. Such attributes may in part correlate with the detection of a relatively large proportion of anthracyclines (>50%) retained intracellularly 24-hours post selective “targeted” delivery [67] where they are found primarily associated with membrane structures or it becomes distributed throughout the cytosol environment [69] [81]. Alternatively, non-covalently bound or “free” anthracycline following passive diffusion across an intact lipid bi-layer membrane is detected primarily within complexes associated with nuclear DNA less than 30 minutes after initial exposure [69]. The anthracycline moiety liberated from covalent immunochemotherapeutics reportedly distributes preferentially into, and accumulates within the nucleus, mitochondria and golgi apparatus [26]. The covalent bonding of gemcitabine to monoclonal immunoglobulin similar to gemcitabine-(C_4_-*amide*)-[anti-EGFR] and gemcitabine-(C_4_-*amide*)-[anti-HER2/*neu*] could therefore function as a molecular strategy for combating patterns of chemotherapeutic resistance in neoplastic cell types. Fortunately, EGFR and HER2/*neu* trophic membrane receptors are both over-expressed in several resistant forms of breast cancer [82]–[84] where their refractory response to chemotherapy is associated with an over-expression of transmembrane P-glycoprotein [85]–[90]. Recognition of these inter-relationships between cancer cell biology and selective “targeted” chemotherapeutic delivery directly correlates with the frequent association between chemotherapeutic-resistance, elevated cancer cell survival parameters, and increased proliferation rates (e.g. relevant to local invasiveness and metastatic dissemination) [91] [92].

Utilization of endogenous trophic membrane receptors that regulate neoplastic cell proliferation and viability as “sites” to facilitate selective “targeted” chemotherapeutic delivery on the exterior surface membrane provides an opportunity to potentially exert cytotoxic properties that are independent of those associated with the chemotherapeutic moiety. Most therapeutic immunoglobulins with binding-avidity for uniquely or highly over-expressed endogenous trophic membrane receptors competitively “block” binding of receptor ligands (e.g. EGF ⇉| IgG::EGFR). Suppression of neoplastic cell growth and vitality is therefore achieved by preventing activation, or inhibit the biological function of EGFR, HER2/*neu*, IGFR, VEGFR and similar trophic membrane receptors that directly or indirectly regulate proliferation kinetics, metastatic behavior and chemotherapeutic-resistance. Similarly, internalization of EGFR, HER2/*neu*, IGFR, VEGFR or analogous endogenous trophic membrane receptors by mechanisms of receptor-mediated-endocytosis promotes transient down-regulation, or partial to complete depletion of their expression resulting in declines in membrane density that lead to suppression of neoplastic cell viability and proliferation rate. In order for this phenomenon to occur trophic membrane receptor “*sites*” must to a variable degree become physically depleted in a manner that is partially due to a deficient rate of re-expression and replenishment to original baseline levels. The rate and extent at which trophic receptor complexes are internalized by immunoglobulin-induced receptor-mediated endocytosis is directly determined by the; [*i*] availability (quantity and concentration) of covalent immunochemotherapeutics; [*ii*] expression density of membrane “sites” utilized to facilitate selective “targeted” chemotherapeutic delivery and progressive membrane deposition; and the [*iii*] corresponding rate and extent that uniquely or highly over-expressed trophic membrane receptors or similar “*sites*” are re-expressed and replenished on the exterior surface of neoplastic cell populations.

Binding of the immunoglobulin component of gemcitabine-(C_4_-*amide*)-[anti-EGFR], gemcitabine-(C_4_-*amide*)-[anti-HER2/*neu*] or any other analogous covalent immunochemotherapeutics at endogenous trophic membrane receptor sites over-expressed on the exterior surface membrane of neoplastic cell types can in an *in-vivo* environment provide additional levels of selective anti-neoplastic cytotoxicity. Such enhancements in selective anti-neoplastic cytotoxicity that are difficult to comprehensively detect *ex-vivo* entail and are dependent upon the recruitment of multiple innate immune responses. Given this perspective, binding of covalent immunochemotherapeutics on the exterior surface membrane of neoplastic cells stimulate or activate; [*i*] complement C9 mediated cytolysis; [*ii*] opsonization secondary to immunoglobulin binding at endogenous trophic membrane receptor sites and subsequent formation of IgG/receptor/complement complexes (e.g. induced macrophage phagocytosis); and [*iii*] antibody-dependent cell-mediated cytotoxicity (ADCC: classically requiring recruitment of NK/natural killer lymphocytes or to a lesser degree participation of macrophages, neutrophils and eosinophils. Collectively these three host immune responses represent the major mechanism of selective anti-neoplastic cytotoxicity evoked by anti-CD20, anti-CD52 and similar monoclonal immunoglobulins utilized for the therapeutic management of haemopoietic neoplasias (e.g. chronic lymphocytic leukemia). Despite the potential for gemcitabine-(C_4_-*amide*)-[anti-EGFR] and gemcitabine-(C_4_-*amide*)-[anti-HER2/*neu*] to collectively stimulate complement C9 mediated lysis, ADCC responses and promote IgG/complement facilitated opsonization of neoplastic cells in a manner that attains enhanced levels of selective anti-neoplastic cytotoxicity, it continues to be technically difficult to simultaneously simulate and accurately measure each of these three immune-dependent responses utilizing *ex-vivo* models for neoplastic disease states.

In clinical scenarios were immunoglobulin fractions are utilized to selectively “target” delivery of therapeutic pharmaceuticals or diagnostic imaging agents in nuclear medicine the antibody component can be biochemically modified with enzyme preparations like papain in order to cleave and remove the Fc segment of the IgG molecule. Biochemical modifications of this type minimize non-selective binding of immunochemotherapeutics to Fc receptors expressed by cell types that comprise the RE system (mononuclear phagocytic system) that anatomically reside within the spleen and liver. Unfortunately, such biochemical modifications create a covalent immunochemotherapeutic composed predominantly of only F(ab′)_2_ or Fab′ that have less of a capacity to activate the complement cascade (e.g. C9 cytolysis, C3b/C4b opsonization), increase neoplastic cell opsonization (e.g. macrophage Fc receptor dependent binding), or promote stimulation of ADCC (e.g. NK lymphocyte Fc receptor dependent binding).

Dual selective “targeted” binding of both gemcitabine-(C_4_-*amide*)-[anti-EGFR] and gemcitabine-(C_4_-*amide*)- [anti-HER2/*neu*] at two different trophic membrane receptors over-expressed (e.g. EGFR) or highly over-expressed (e.g. HER2/*neu*) on a single neoplastic cell type provides a range of opportunities for achieving greater levels of anti-neoplastic cytotoxicity than is possible with only a single covalent gemcitabine immunochemotherapeutic. Gemcitabine-(C_4_-*amide*)-[anti-EGFR] in dual-combination with gemcitabine-(C_4_-*amide*)-[anti-HER2/*neu*] therefore can provide heightened planes of anti-neoplastic cytotoxicity through several molecular strategies.

### Level-1

Greater concentrations of selectively “targeted” chemotherapeutic concentrations within the cytosol of neoplastic cell populations that presents a potential opportunity for resolving neoplastic cell types that are partially resistant when the “parent” conventional chemotherapeutic is administered intravenously at clinically relevant and safe dosages. Dual selective “targeted” chemotherapeutic delivery also represents a strategy for combating chemotherapeutic resistance as can occur with alterations in P-glycoprotein expression [82]–[84].

### Level-2

Dual simultaneous inhibition of the biological functions and properties of multiple both endogenous trophic membrane receptors or other sites over-expressed on the exterior surface chemotherapeutic-resistant mammary adenocarcinoma (SKBr-3). Synergistic levels of anti-neoplastic cytotoxicity achieved solely through inhibition of multiple endogenous trophic membrane receptors or analogous biological “targets” can only theoretically be achieved if each different site has distinctly different biological functions/properties within a given neoplastic cell type (e.g. EGFR-*vs*-HER2/*neu* or CD20*-vs-*CD74) [93] [94].

### Level-3

Selectively “targeted” gemcitabine delivery in dual and simultaneous combination with inhibition of trophic membrane receptor function represents an opportunity for imposing a distinct plane of additive or synergistic anti-neoplastic cytotoxicity especially when trophic receptor “targets” are over-expressed (e.g. SKBr-3: EGFR) or highly over-expressed (e.g. SKBr-3: HER2/*neu*) [13] [14] [95]–[104]. Additive or synergistic interactions of this type have been detected between anti-HER2/*neu* applied in simultaneous combination with cyclophosphamide [14] [95], docetaxel [95], doxorubicin [14] [95], etoposide [95], methotrexate [95], paclitaxel [14] [95], or vinblastine [95]. The dual-combination of gemcitabine-(C_4_-*amide*)-[anti-EGFR] with gemcitabine-(C_4_-*amide*)-[anti-HER2/*neu*] therefore provides two potential avenues for achieving additive and synergistic levels of cytotoxicity exerted by gemcitabine chemotherapeutic and each anti-trophic receptor immunoglobulin fractions (e.g. gemcitabine with anti-EGFR; gemcitabine with anti-HER2/*neu*; gemcitabine with anti-EGFR and anti-HER2/*neu*).

### Level-4

Simultaneous binding of covalent immunochemotherapeutic combinations like gemcitabine-(C_4_-*amide*)-[anti-EGFR] and gemcitabine-(C_4_-*amide*)-[anti-HER2/*neu*] at two different over-expressed trophic receptor types on the exterior surface membrane of a single cancer cell population *in-vivo* offers the potential to attain a third plane of additive and synergistic anti-neoplastic cytotoxicity from innate immune response mechanisms. Selectively “targeted” additive or synergistic anti-neoplastic cytotoxicity can potentially occur *in-vivo* through the different combined properties of; [*i*] complement C9 mediated cytolysis; [*ii*] IgG/receptor/complement-facilitated opsonization; and [*iii*] IgG-dependent cell-mediated cytotoxicity (ADCC). Conceptually, at least, the simultaneous binding of gemcitabine-(C_4_-*amide*)-[anti-EGFR] and gemcitabine-(C_4_-*amide*)-[anti-HER 2/*neu*] at two different endogenous trophic receptors on the same cancer cell type offers the probability of evoking a greater degree of selectively “targeted” anti-neoplastic cytotoxicity compared to the selective binding of just a single covalent gemcitabine immunochemotherapeutic.

### Level-5

Dual simultaneous combinations of gemcitabine-(C_4_-*amide*)-[anti-EGFR] and gemcitabine-(C_4_-*amide*)-[anti-HER2/*neu*] *in-vivo* presents an opportunity to potentially attain still another plane of additive and synergistic anti-neoplastic cytotoxicity that involves; [*i*] gemcitabine in dual-combination with innate immune responses; [*ii*] trophic receptor inhibition in dual-combination with innate immune responses; and/or [iii] gemcitabine, trophic receptor inhibition and innate immune responses. In support of this concept, immune cell populations that are involved in ADCC phenomenon release cytotoxic components known to additively and synergistically enhance the cytotoxic anti-neoplastic activity of conventional chemotherapeutic agents [105]. Undoubtedly, other immune responses also contribute to the anti-neoplastic properties of many conventional chemotherapeutic agents. Recognition of the phenomenon where different immune-dependent responses become a significant component of additive and synergistic anti-neoplastic cytotoxicity phenomenon in active partnership with chemotherapeutic moieties and trophic receptor inhibition at least in part delineates how covalent immunochemotherapeutics frequently evoke greater efficacy when implemented *in-vivo* compared to levels of anti-neoplastic cytotoxicity observed utilizing *ex-vivo* based models for neoplastic disease even when the same identical cancer cell types (xenographs) are utilized [106]–[108]. Each of the qualities and properties discussed for the selective “targeted” chemotherapeutic delivery and additive or synergistic interactions that can be evoked by gemcitabine-(C_4_-*amide*)-[anti-EGFR] and gemcitabine-(C_4_-*amide*)-[anti-HER2/*neu*] collectively serve to explain how the dual-combination of these two covalent immunochemotherapeutics produced additive levels of anti-neoplastic cytotoxicity measured in chemotherapeutic-resistant mammary-adenocarcinoma (SKBr-3) populations functioning as an *ex-vivo* model for neoplastic disease (Figure 8). In part, the basis for this perception originates from the observation that when gemcitabine-(C_4_-*amide*)-[anti-EGFR] and gemcitabine-(C_4_-*amide*)-[anti-HER2/*neu*] were formulated as a 50:50 gemcitabine-equivalent combination the levels of anti-neoplastic cytotoxicity detected were intermediate between each of the two individual gemcitabine immunochemotherapeutics formulated at gemcitabine-equivalent concentrations (Figure 8).

Several variables related to methods and techniques could be have been modified to increase and maximize the anti-neoplastic cytotoxicity of gemcitabine-(C_4_-*amide*)-[anti-EGFR] in dual simultaneous combination with gemcitabine-(C_4_-*amide*)-[anti-HER2/*neu*].

- Almost invariably, levels of anti-neoplastic cytotoxicity can be increased by prolonging the *ex-vivo* incubation period during which time neoplastic cell populations are challenged in direct and simultaneous contact with each of the two covalent gemcitabine immunochemotherapeutics.
- A human neoplastic cell type other than chemotherapeutic-resistant mammary adenocarcinoma (SKBr-3) could have been applied to access anti-neoplastic cytotoxicity of gemcitabine-(C_4_-*amide*)-[anti-EGFR] in dual simultaneous combination with gemcitabine-(C_4_-*amide*)-[anti-HER2/*neu*]. Similarly, human mammary carci-noma (MCF-7/WT-2′) [61] and mammary adenocarcinoma (BG-1) [61] are both known to be relatively resistant to gemcitabine and covalent gemcitabine-(oxyether phopholipid). The two covalent gemcitabine immunochemotherapeutics likely would have evoked greater levels of anti-neoplastic cytotoxicity if it had been measured utilizing populations of pancreatic carcinoma, [109] small-cell lung carcinoma, [110] neuroblastoma, [111] or leukemia/lymphoma [61] [112] because of their relatively higher gemcitabine sensitivity. Similarly, human promyelocytic leukemia, [59] [61] T-4 lymphoblastoid clones, [61] glioblastoma; [59] [61] cervical epitheliod carcinoma, [61] colon adenocarcinoma, [61] pancreatic adenocarcinoma, [61] pulmonary adenocarcinoma, [61] oral squamous cell carcinoma, [61] and prostatic carcinoma [40] have been found to be sensitive to gemcitabine and gemcitabine-(oxyether phopholipid) covalent chemotherapeutic conjugates.
- Analogous to the consideration that gemcitabine-(C_4_-*amide*)-[anti-EGFR] in dual simultaneous combination with gemcitabine-(C_4_-*amide*)-[anti-HER2/*neu*] would have evoked higher levels of anti-neoplastic cytotoxicity in a different neoplastic cell type specifically sensitive to gemcitabine, the effectiveness of these two covalent gemcitabine immunochemotherapeutics would likely of been higher in neoplastic cell types not displaying profiles of general chemotherapeutic resistance. Majority of the covalent immunochemotherapeutics described in publications to date have measured cytotoxic efficacy utilizing human neoplastic cell populations that are not chemotherapeutic-resistant. Rare exceptions have been the application of chemotherapeutic-resistant metastatic melanoma M21 (covalent daunorubicin immunochemotherapeutics synthesized using anti-chondroitin sulfate proteoglycan 9.2.27 surface marker); [63] [64] [113] chemotherapeutic-resistant mammary carcinoma MCF-7AdrR (covalent anthracycline-ligand chemotherapeutics utilizing epidermal growth factor (EGF) or an EDF fragment); [114] and chemotherapeutic-resistant mammary adenocarcinoma (SKBr-3) populations (epirubicin-anti-HER2/*neu*, [16] [18] [25] epirubicin-anti-EGFR, [16] gemcitabine-HER2/*neu* [19] [24]) respectively.
- Assessment of neoplastic cellular proliferation with either [^3^H]-thymidine, or an ATP-based assay method would likely have resulted in recognizing lower degrees of early anti-neoplastic cytotoxicity because these analytical modalities reportedly are ≥10-fold more sensitive in detecting lower degrees of early sub-lethal anti-neoplastic cytotoxicity compared to MTT vitality stain based assay methods [115] [116]. In spite of this perception, MTT vitality stain based assays continue to be extensively applied for the routine assessment of true anti-neoplastic cytotoxicity of chemotherapeutics covalently incorporated synthetically into molecular platforms that provide properties of selective “targeted” delivery [16] [59]–[61] [117]–[122]. One notable and significant advantage of MTT vitality stain based assay and other methods applying similar reagents is their ability to indirectly detect and measure lethal anti-neoplastic cytotoxic potency. Potency measured *ex-vivo* In this manner is generally considered to be superior to merely detecting early-stage sub-lethal cellular injury that could potentially be reversible and be more difficult to correlated with *in-vivo* levels of efficacy and potency.
- Lastly, as previously eluded to, the anti-neoplastic cytotoxicity of the dual simultaneous combination of the covalent gemcitabine immunochemotherapeutics, gemcitabine-(C_4_-*amide*)-[anti-EGFR] and gemcitabine-(C_4_-*amide*)-[anti-HER2/*neu*] would likely have been greater if their efficacy had been delineated in an *in-vivo* model for cancer such as human neoplastic xenographs in animal hosts. In such neoplastic disease models, the added effect of host immune responses in the form of selectively “targeted” antibody-dependent cell cytotoxicity (ADCC), complement C9 mediated cytolysis, and/or opsonization/phagocytosis would have been realized.

Levels of anti-neoplastic cytotoxicity vary between different organoselenium compounds when assessed independently as a single agent or in combination with conventional chemotherapeutics [123]–[127]. Various forms of selenium have been reported to additively or synergistically complement the anti-neoplastic cytotoxicity of anthracyclines [123]–[126] [128], irinotecan [127] [129]–[131], docetaxel/paclitaxel [124] [132], and tamoxifen [133]. In the presence of selenium the vulnerability of B-cell lymphoma to the anti-neoplastic cytotoxicity of doxorubicin, etoposide, 4-hydroxyperoxycyclophosphamide, melphalan, and 1-*β*-*D*-arabinofuranosyl-cytosine increases approximately 2.5-fold (e.g. methylseleninate 10 – 100 μM) [134]. Synergism achieved with selenium in dual-combination with conventional chemotherapeutics can ultimately become additive during prolonged periods of challenge (incubation) or when the duration of clinical administration and treatment is extended [128]. Interestingly, selenium exerts greater cytotoxic anti-neoplastic activity compared to celecoxib [135]–[139] when analyzed at micromolar equivalent concentrations.

Selenium can potentially bestow therapeutically beneficial properties through induction of a number of biological effects or responses in neoplastic cell populations such as its capacity to; [*i*] induce apoptosis in doxorubicin-resistant lung small-cell carcinoma (selenite 10 μM) [140]; [*ii*] promote severe ER stress (leukemia cell types) [141]; and [*iii*] reduce vitality of multidrug-resistant leukemia (selenite-triglycerides 10 μg to 40 μg/ml) [142]. Several specific molecular mechanisms explain the anti-neoplastic cytotoxicity induced by selenium. Selenium (selenite) causes cell death through activation of the pro-apoptotic transcription factor GADD153 and high concentrations in leukemia cells promote p53 activation [141]. Selenium (selenite) independently mediates anti-neoplastic activity through p53 activation and increased oxidative stress which collectively precipitate mitochondrial dysfunction and caspase activation (leukemia cell types) [141]. Elevated levels of oxidative stress occur at relatively high selenium concentrations [141] which is accompanied by, or a direct result of reductions in catalase enzyme activity (H_2_O_2_ → H_2_O + O_2_) [123]. In addition to the influence of selenium on caspase activation, it also promotes apoptosis by increasing Fas-associated death domain (FADD) expression and enhancement of caspase-8 recruitment for Fas and FADD (MCF7 breast cancer) [126]. Selenium is believed to trigger apoptosis by additionally increasing FOXO3a transcriptional factor activity [125] that occurs in concert with Bim [125] and PUMA up-regulation, or alternatively the down-regulation of FLIP anti-apoptotic protein. Gradient increases in selenium concentrations (1 μM to 10 μM) induce dose-dependent elevations in the amount and activity of thioredoxin reductase in non-resistant neoplastic cells while precipitating declines in thioredoxin reductase (e.g. doxorubicin-resistant small cell carcinoma) [140]. Thioredoxin reductase biochemically reduces thioredoxin which mediates the final step of the electron-transfer pathway for nucleoside diphosphate reduction where in cancer cells is essential for cell growth and survival.

In populations of chemotherapeutic-resistant mammary adenocarcinoma (SKBr-3), methylseleninate [125] [126] had greater anti-neoplastic cytotoxic potency than [Se]-methylselenocysteine [129] [133] at selenium-equivalent concentrations of 10 μM and 20 μM but they were similar at the selenium-equivalent concentrations of 30 μM, 40 μM and 50 μM (Figure 9). Selenium also can potentially contribute to the efficacy of conventional small molecular weight chemotherapeutic agents. In chemotherapeutic-resistant mammary adenocarcinoma (MCF-7) selenium increases sensitivity to anthracyclines [125] and in combination with doxorubicin it influences Fas signaling [126] at methylseleninate concentrations of 2.5 μM and 5 μM respectively. Selenium also increases mitochondrial caspase-9 activation which promotes apoptosis and produces synergistic levels of anti-neoplastic cytotoxicity in combination with anthracyclines (e.g. mammary adenocarcinoma MCF7 cell type). [126] Analogous investigations determined that selenium in the form of methylseleninate also complements the anti-neoplastic cytotoxic efficacy of selectively “targeted” covalent immunochemotherapeutics including epirubicin-(C_13_-*imino*)-[anti-HER2/*neu*] [25]. The organoselenium agent, [Se]-methylselenocysteine in preference to methylseleninate was evaluated to determine if it could complement the anti-neoplastic cytotoxicity of the two covalent gemcitabine immunochemotherapeutics applied in dual simultaneous combination because it was considered more suitable for *in-vivo* administration (Figure 9 and Figure 10). Total anti-neoplastic cytotoxicity of gemcitabine-(C_4_-*amide*)-[anti-EGFR] in dual simultaneous combination with gemcitabine-(C_4_-*amide*)-[anti-HER2/*neu*] increased substantially in the presence of [Se]-methylselenocysteine formulated at a fixed 15 μM selenium-equivalent concentration (Figure 10). Increases in total anti-neoplastic cytotoxicity for gemcitabine-(C_4_-*amide*)-[anti-EGFR] and gemcitabine-(C_4_-*amide*)-[anti-HER2/*neu*] when utilized in concert with [Se]-methylselenocysteine was most prominent at and between the gemcitabine-equivalent concentrations of 10^−10^ M and 10^−7^ M but was nearly equivalent at 10^−6^ M (Figure 10). Similar to *α*-tocopherol, one property of selenium that may be particular beneficial for improving the efficacy and potency of covalent immunochemotherapeutics or covalent [receptor ligand]-chemotherapeutics with binding-avidity for over-expressed endogenous membrane receptor sites is an ability to potentially improve their internalization by mechanisms of receptor-mediated-endocytosis [143]. Such claims are however somewhat speculative since they are based on the observation that selenium and *α*-tocopherol deficiencies reduce receptor-mediated processes possibly associated with greater levels of membrane oxidation and alterations in membrane fluidity.

**Figure 11:**
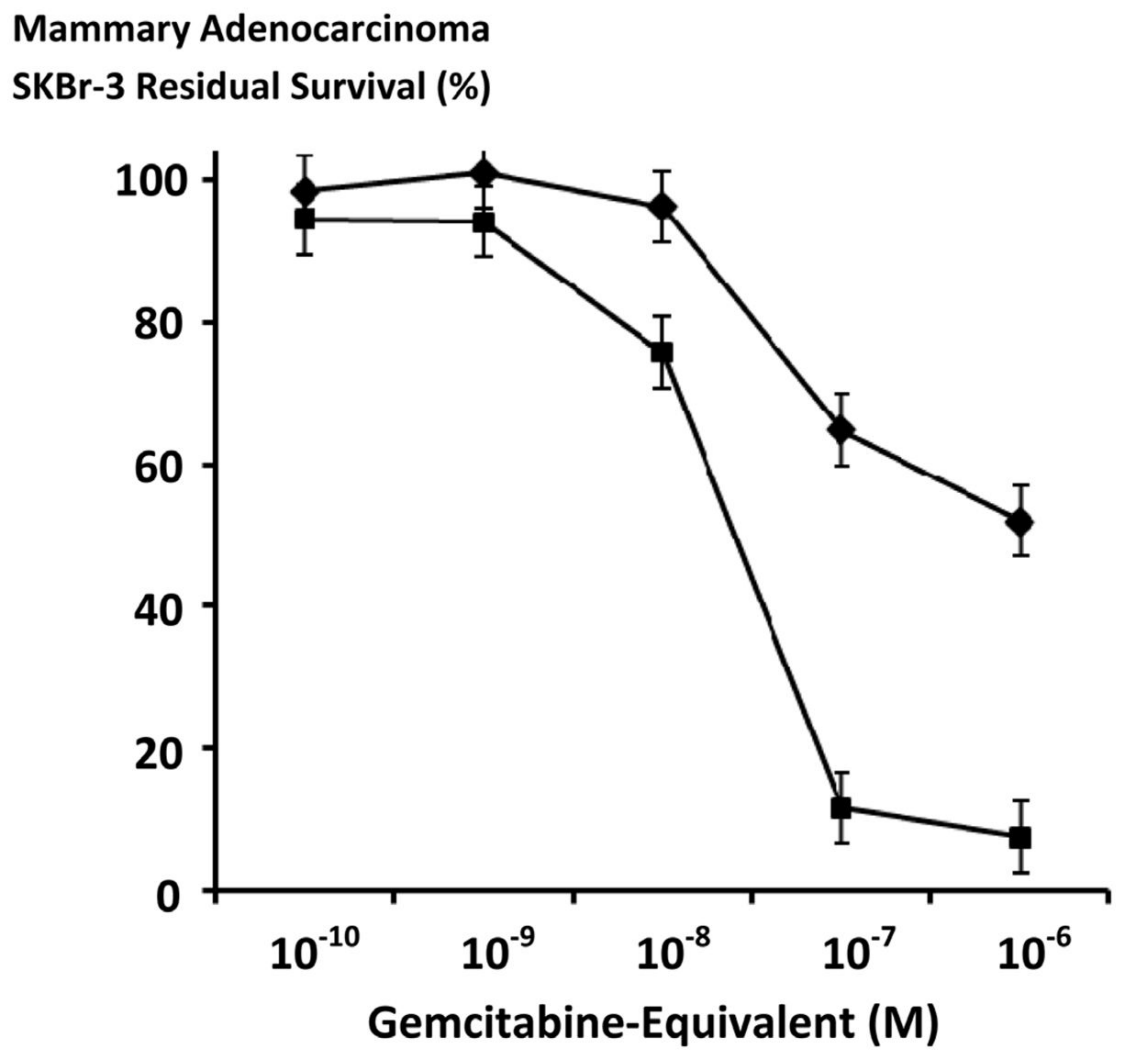
Relative gemcitabine anti-neoplastic cytotoxicity against chemotherapeutic-resistant mammary adenocarcinoma over challenge (incubation) periods of different duration. *Legends*: (◆) gemcitabine following a 96-hour incubation period; and (■) gemcitabine following a 182-hour incubation period. Gemcitabine formulated in triplicate at gradient gemcitabine-equivalent concentrations was incubated in direct contact with triplicate populations of chemotherapeutic-resistant mammary adenocarcinoma (SKBr-3) during incubation periods of 96-hours or 182-hours. Anti-neoplastic cytotoxicity was measured using a MTT cell vitality assay relative to matched negative reference controls.

Interpretation of the anti-neoplastic cytotoxicity analysis of organoselenium analogs in the form of [Se]-methylselenocysteine and methylseleninate suggests that they could be used to achieve specific levels of anti-neoplastic cytotoxicity at lower total gemcitabine-equivalent concentrations of gemcitabine-(C_4_-*amide*)-[anti-EGFR], gemcitabine-(C_4_-*amide*)-[anti-HER2/*neu*] or gemcitabine (Figures 9–11). Conservative extrapolation from this observed result implies that organoselenium compounds when applied in dual simultaneous combination with gemcitabine or covalent gemcitabine immunochemotherapeutics could provide an opportunity for achieving more complete and more rapid resolution of neoplastic conditions while also lowering total chemotherapeutic dosage requirements in a manner that would produce fewer serious side-effects or sequelae.

## 5. Conclusions

The covalent immunochemotherapeutics, gemcitabine-(C_4_-*amide*)-[anti-EGFR] and gemcitabine-(C_4_-*amide*)-[anti-HER2/*neu*] each have potent selective “targeted” anti-neoplastic cytotoxic properties against chemotherapeutic-resistant mammary adenocarcinoma (SKBr-3). Applied in dual simultaneous 50/50 combination, gemci-tabine-(C_4_-*amide*)-[anti-EGFR] and gemcitabine-(C_4_-*amide*)-[anti-HER2/*neu*] collectively evoke levels of selective “targeted” anti-neoplastic cytotoxicity that were intermediate between chemotherapeutic-equivalent concentrations of gemcitabine-(C_4_-*amide*)-[anti-EGFR] and gemcitabine-(C_4_-*amide*)-[anti-HER2/*neu*]. Vitality/viability profiles for chemotherapeutic-resistant mammary adenocarcinoma were compatible with the concept that gemcitabine was internalized independently but simultaneously at two separate endogenous trophic membrane receptor sites (e.g. EGFR, HER2/*neu*) by mechanisms of receptor-mediated endocytosis. Simultaneous dual selective “targeted” delivery of gemcitabine facilitated by gemcitabine-(C_4_-*amide*)-[anti-EGFR] and gemcitabine-(C_4_-*amide*)-[anti-HER2/*neu*] therefore serves as a prototype molecular strategy for maximizing cytosol chemotherapeutic concentrations selectively within a given neoplastic cell type. In this context the anti-EGFR and anti-HER2/*neu* immunoglobulin components of gemcitabine-(C_4_-*amide*)-[anti-EGFR] and gemcitabine-(C_4_-*amide*)-[anti-HER2/*neu*] promote [*i*] selective “targeted” gemcitabine delivery; [*ii*] progressive gemcitabine deposition on the exterior surface membrane of neoplastic cells; and [*iii*] accumulation of the gemcitabine moiety within the cytosol to concentrations that are far beyond levels attainable by simple passive diffusion following IV infusion of clinically relevant and safe dosages. While simultaneous selective “targeted” delivery of gemcitabine by gemcitabine-(C_4_-*amide*)-[anti-EGFR] applied in dual-combination with gemcitabine-(C_4_-*amide*)-[anti-HER2/*neu*] can potentially improve the resolution of neoplastic disease states, such benefits would in part be made possible through reduction in innocent chemotherapeutic exposure by healthy tissues and normal organ systems.

Selenium in the form of [Se]-methylselenocysteine and methylseleninate both demonstrated anti-neoplastic cytotoxicity against chemotherapeutic-resistant mammary adenocarcinoma (SKBr-3). Applied in concert with the dual simultaneous combination of gemcitabine-(C_4_-*amide*)-[anti-EGFR] and gemcitabine-(C_4_-*amide*)-[anti-HER2/*neu*], the anti-neoplastic cytotoxicity attained was substantially greater in the presence of [Se]-methylselenocysteine. Collectively, research investigations with gemcitabine-(C_4_-*amide*)-[anti-EGFR], gemcitabine-(C_4_-*amide*)-[anti-HER2/*neu*] and selenium at least in the form of [Se]-methylselenocysteine demonstrate therapeutic options that may be more effective in resolving chemotherapeutic-resistant neoplastic conditions within a more expedient treatment time frame implementing lower total dosage levels.
